# Anthropogenic landscape decreases mosquito biodiversity and drives malaria vector proliferation in the Amazon rainforest

**DOI:** 10.1371/journal.pone.0245087

**Published:** 2021-01-14

**Authors:** Leonardo Suveges Moreira Chaves, Eduardo Sterlino Bergo, Jan E. Conn, Gabriel Zorello Laporta, Paula Ribeiro Prist, Maria Anice Mureb Sallum

**Affiliations:** 1 Departamento de Epidemiologia, Faculdade de Saúde Pública, Universidade de São Paulo, São Paulo, SP, Brazil; 2 Superintendência de Controle de Endemias, Secretaria de Estado da Saúde de São Paulo, Araraquara, SP, Brazil; 3 Wadsworth Center, New York State Department of Health, Albany, NY, United States of America; 4 Department of Biomedical Sciences, School of Public Health, State University of New York, Albany, NY, United States of America; 5 Setor de Pós-graduação, Pesquisa e Inovação, Centro Universitário Saúde ABC, Fundação ABC, Santo André, SP, Brazil; 6 Department of Ecology, Institute of Bioscience, University of São Paulo, São Paulo, SP, Brazil; Universidade Estadual de Santa Cruz - UESC, BRAZIL

## Abstract

Inter-relationships among mosquito vectors, *Plasmodium* parasites, human ecology, and biotic and abiotic factors, drive malaria risk. Specifically, rural landscapes shaped by human activities have a great potential to increase the abundance of malaria vectors, putting many vulnerable people at risk. Understanding at which point the abundance of vectors increases in the landscape can help to design policies and interventions for effective and sustainable control. Using a dataset of adult female mosquitoes collected at 79 sites in malaria endemic areas in the Brazilian Amazon, this study aimed to (1) verify the association among forest cover percentage (PLAND), forest edge density (ED), and variation in mosquito diversity; and to (2) test the hypothesis of an association between landscape structure (i.e., PLAND and ED) and *Nyssorhynchus darlingi* (Root) dominance. Mosquito collections were performed employing human landing catch (HLC) (peridomestic habitat) and Shannon trap combined with HLC (forest fringe habitat). *Nyssorhynchus darlingi* abundance was used as the response variable in a generalized linear mixed model, and the Shannon diversity index (H’) of the Culicidae community, PLAND, and the distance house-water drainage were used as predictors. Three ED categories were also used as random effects. A path analysis was used to understand comparative strengths of direct and indirect relationships among Amazon vegetation classes, Culicidae community, and *Ny*. *darlingi* abundance. Our results demonstrate that *Ny*. *darlingi* is negatively affected by H´ and PLAND of peridomestic habitat, and that increasing these variables (one-unit value at β_0_ = 768) leads to a decrease of 226 (*P* < 0.001) and 533 (*P* = 0.003) individuals, respectively. At the forest fringe, a similar result was found for H’ (β_1_ = -218; *P* < 0.001) and PLAND (β_1_ = -337; *P* = 0.04). Anthropogenic changes in the Amazon vegetation classes decreased mosquito biodiversity, leading to increased *Ny*. *darlingi* abundance. Changes in landscape structure, specifically decreases in PLAND and increases in ED, led to *Ny*. *darlingi* becoming the dominant species, increasing malaria risk. Ecological mechanisms involving changes in landscape and mosquito species composition can help to understand changes in the epidemiology of malaria.

## Introduction

Various interconnected mechanisms associated with deforestation and land-use, such as environmental change, abundance and size of deforested patches, and biodiversity loss can alter human exposure to many infectious diseases, especially malaria [1–5]. The relationship between biodiversity and its capacity to protect humans from infectious diseases has been widely debated and, as suggested by Johnson et al. [6], the central discussion is how ecological interactions among hosts, parasites and environmental factors change diversity and disease risk.

The dilution effect hypothesis (DEH) supports the premise that high biodiversity dilutes the infection sources represented by reservoir species and reduces disease risk [7, 8]. As a corollary to the DEH, pathogen competent hosts are frequently found in low diversity settings [9–11]. The diffuse competition hypothesis (DCH), posits that the high diversity of non-vector mosquitoes blood feeding on a few vertebrate species may increase the defensive behavior of the host, thus decreasing the number of bites and the risk of exposure to infective vectors [12, 13]. Laporta et al. [3] found a strong association among reduced malaria risk, increased diversity of the mosquito community caused by the augmentation in DCH and high diversity of sylvatic vertebrates, linked to increased DEH.

The DEH was supported in an empirical test of mosquito-borne diseases in Panama [14]. In the malaria endemic Amazon basin, the dilution effect is highly heterogeneous due in part to differential drivers of human behavior, economic, cultural, and social factors, which can influence malaria risk [15, 16]. The *Plasmodium* life cycle requires highly specific inter-relationships among humans, mosquitoes, and parasites [17]. We propose that anthropogenic changes in a native forest environment, such as forest fragmentation, will modify patterns of *Plasmodium* parasite activity, representing expansion of a malaria risk area, and a challenge for malaria control.

Forest fragmentation increases ecotone habitats [18, 19], causing changes in the spatiotemporal abundance of anopheline larval habitats [20–23], increased vector abundance [24–27] and malaria risk [4, 28–31]. According to the deforestation-malaria link, defined by Sawyer [32] and de Castro et al. [16] as “frontier malaria”, forest clearing increases the average temperature in agricultural settlements and in neighboring larval habitats. Temperature increase can lead to a decrease in the larva to adult development time, increase in adult survivorship and in vectorial capacity of mosquito populations [21, 33–35].

Moreover, economic development is associated with the exploitation of forest commodities and timber, agriculture, and cattle ranch expansion [36], and as such, land occupation requires continuous human movement within the region. Such human movement promotes propagation of *Plasmodium* to new areas, and persistence in others where the disease is decreasing, as demonstrated in Burkina Faso, Africa [37]. The dynamics of socioecological change, poor housing, inadequate access to water and sanitation, and lack of or poor access to health facilities by the inhabitants also serve to intensify malaria transmission [38].

*Nyssorhynchus darlingi* (Root) is the primary vector in Amazonian Brazil [39, 40]. Both the occurrence and increased abundance of this species in malaria landscapes have been associated with spatial and temporal distribution of aquatic habitats for female oviposition and the development of immature stages [20, 41]. Aquatic habitats include anthropogenic and natural water bodies such as freshwater streams, small forest rivers (igarapés), and partially shaded fishponds, usually located along the forest border [34]. Continuous changes in the landscape also influence the spatiotemporal dynamics of the malaria vectors, that normally exhibit seasonal peaks in abundance depending on the environmental distribution and availability of habitats in the wet-dry and dry-wet transition seasons [42–44]. Anthropogenic changes in natural environments either can increase the distribution and availability of standing water despite season or increase the duration of habitats, thus diminishing the seasonal impact on the dynamics of vector populations (Rohr et al. 2019).

Culicidae diversity and landscape composition or configuration, distinct patterns of land occupation and land-use have all been considered important drivers of malaria, changing the biodiversity of ecosystem services [3], shifting primary vector species [24, 45, 46], and exposing the human population to risk [47]. In endemic areas, a decrease in Anophelinae diversity increases both the risk of human exposure to vector mosquitoes and the likelihood of acquiring malaria, especially in areas where the landscape structure is conducive to vector-human contact [24, 27, 48]. In Amazonian rural landscapes, malaria risk is dynamic and associated with a complex network of environmental changes, ecological, entomological, socioeconomic, and political determinants [20, 49, 50].

The source-sink dynamics concept was originally proposed to describe variation in habitat quality that can affect population growth or decline for a target species [51]. More recently, this concept was embraced by the field of landscape ecology [52] and has joined the theory of island biogeography. Immigration and extinction rates across a heterogenous habitat composed of continuous forest (source) and small forest patches (sinks) in an anthropogenic matrix define the persistence of a given species. In the case of mosquitoes, the source-sink dynamics can be applied to understand and predict transmission of sylvatic pathogens to humans, or vice-versa.

This study aims to better understand the effects of change in the diversity, community structure and composition of mosquito species in a continuous range of landscape elements, i.e., percent forest cover and forest edge density configurations, in endemic malaria areas in Amazonian Brazil. We hypothesized that (1) decreased forest cover and increased forest edge density lead to a change in mosquito diversity; (2) *Ny*. *darlingi* dominance is dynamic and highest in areas where forest cover is actively being decreased by human occupation.

## Methods

### Study area

Mosquitoes were collected in 79 sampling units in 12 municipalities in the Brazilian Amazon states of Acre (twice in Acrelândia, Cruzeiro do Sul, Mâncio Lima, Rodrigues Alves), Amazonas (Itacoatiara, Guajará, Humaitá, Lábrea, São Gabriel da Cachoeira), Pará (Pacajá), and Rondônia (Machadinho D’Oeste) (S1 Fig in S1 File). The selection of field localities for conducting mosquito collections is the same protocol described by Sallum et al. [53].

**Fig 1:**
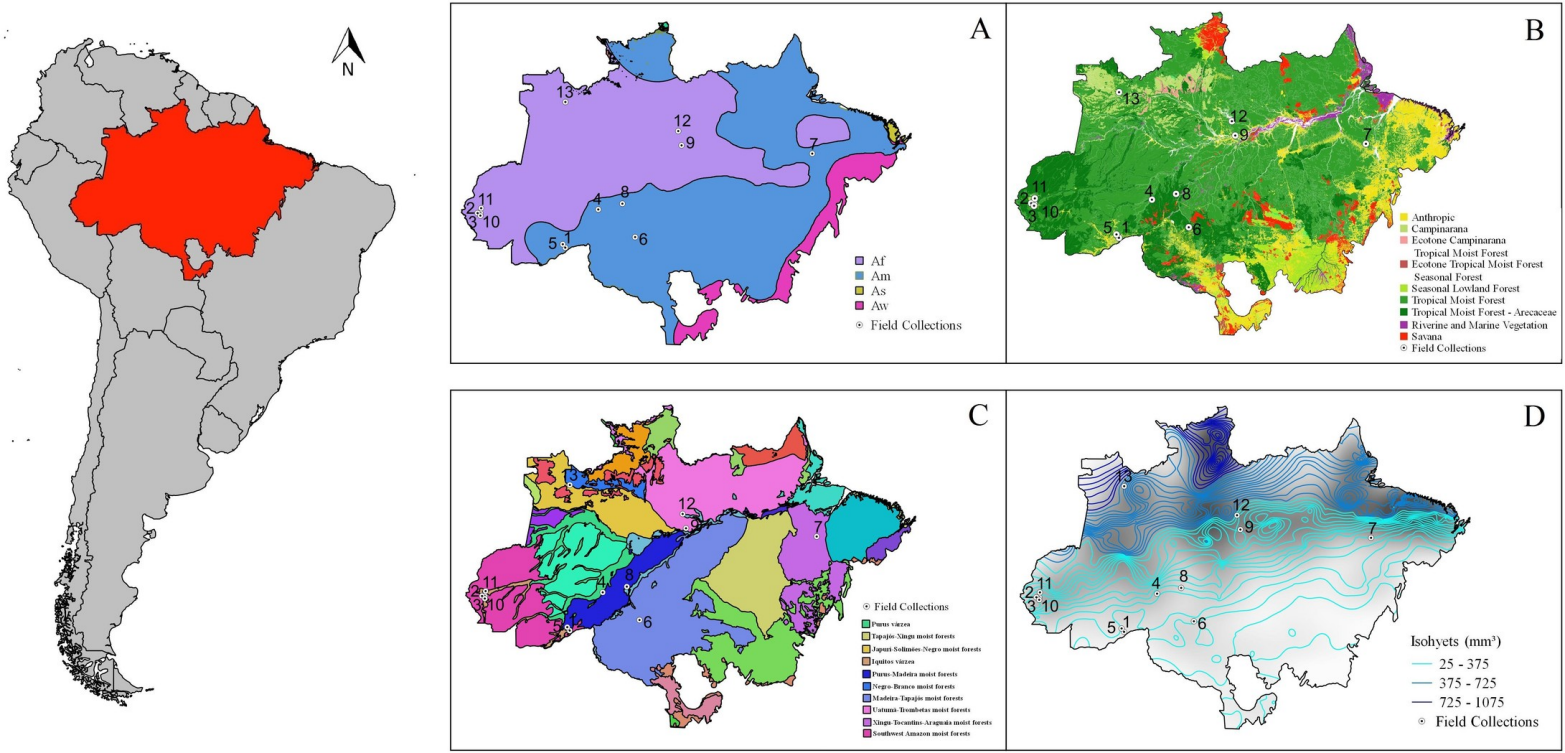
Map of Brazilian Amazonian biome (in red) in South America. A: Köppen and Geiger Climate Classification; Af–Tropical Rainforest, Am–Tropical Monsoon, As–Tropical wet and dry, Aw–Savanna, Cfa–Humid subtropical, Cwa–Subtropical-dry winter. B: Brazilian Vegetation Class (IBGE, 2012); Anthropogenic–dominance of Poaceae, Verbanaceae, Lamiaceae, Solanaceae and Asteraceae botanical families; Tropical Moist Forest–dominance of Lecythidaceae and Vochysiaceae; Tropical Moist Forest Arecaceae–dominance of Lecythidaceae, Vochysiaceae and Arecaceae; Riverine and Marine Vegetation–Arecaceae, Solanaceae, Myrtaceae and Rhizophoraceae; Campinarama–Euphorbiaceae, Arecaceae, Humiriaceae and Fabaceae; Savana–Poaceae, Cyperaceae, Amarylidaceae and Xyridaceae; Seasonal Lowland Forest–Lauraceae, Lythraceae, Boraginaceae, Fabaceae and Myrtaceae. C: Amazonian ecoregions based on biogeography (https://ecoregions2017.appspot.com/) [100]. D: Quarterly Totals Precipitation (mm^3^) of June, July and August from 1977 to 2006 (http://cprm.gov.br). Reprinted from QGIS version 2.8 without any changes, under a CC BY license, with permission from PLOS ONE, original copyright 2020.

Rural settlements comprised mainly subsistence farms, with *Manihot esculenta* (manioc, cassava), *Dioscorea trifida* (yam), *Coffea arabica* (coffee), *Euterpe oleracea* (açaí), *Musa* spp. (banana) and *Oryza* sp. (rice) plantations, and other vegetables for local consumption [54]. Local settlers grow fish in fish farms, and maintain small numbers of poultry, pigs, goats, and livestock for family consumption and local commerce. Details of regional climate, vegetation class, ecological zone, and rainfall are presented in S1 Table in S1 File and Fig 1. The Brazilian Amazon encompasses four major vegetation class: Tropical Moist Forest, Open Tropical Moist Forest, Seasonal Lowland Forest, and Campinarana [55].

**Fig 2:**
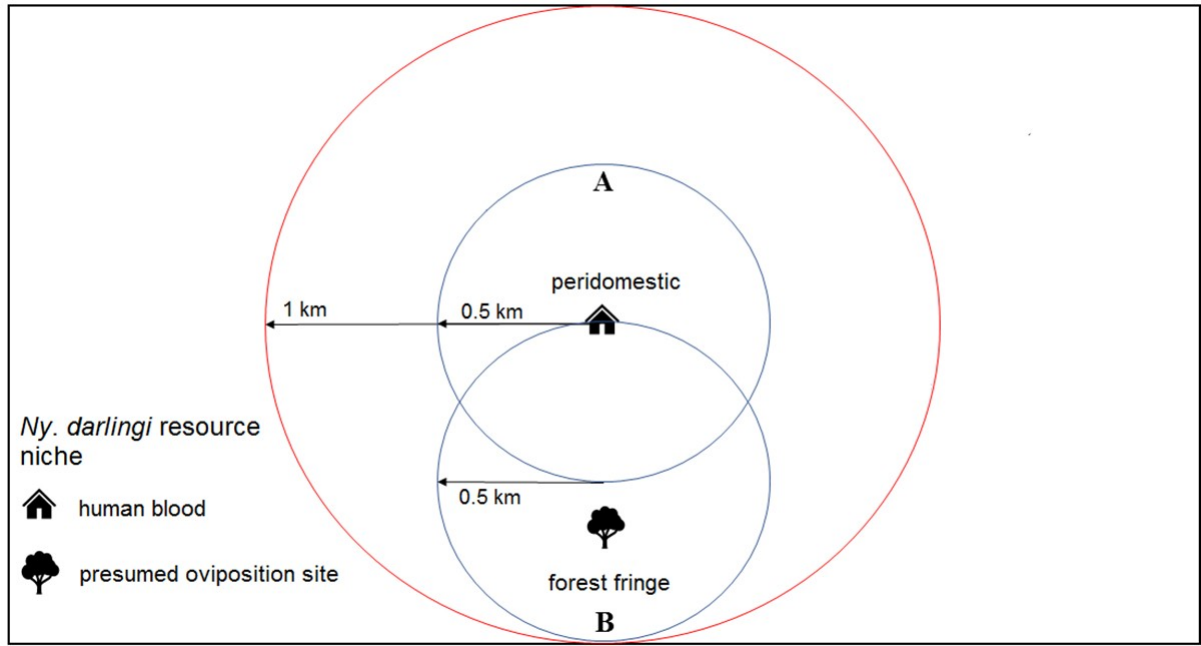
Study design scheme. Large circle (red) represents the sampling unit, and smaller circles (blue) the collection points. A: peridomestic habitat (HLC), and B: forest fringe habitat (ST).

Each of the 79 sampling units was centered on a house around which was subtended a circle of 1 km radius (3.1 km^2^). Each unit consisted of one peridomestic habitat and one forest edge habitat (Fig 2) and was separated from all other units by approximately 2.25 km [56]. There were six sampling units per locality, except for São Gabriel da Cachoeira, Amazonas state, which had seven.

### Mosquito collection

Collections were conducted outdoors in the peridomestic habitat (approximately five meters from the household entrance) using human landing catch (HLC) and in the forest fringe habitat (approximately 10 meters into the forest interface with open areas) with Shannon trap using light and human attraction (ST) [57]. There were two collection points for each sampling unit and HLC and ST collections were conducted simultaneously. Mosquito data from HLC and ST were summed and employed as the field data representative for each sampling unit. Therefore, the total of mosquitoes collected on HLC and ST were evaluated together and used as fixed variable in the models at 1 km radius for each sampling unit.

Sampling was performed once between 18:00 and 0:00h, from April to November 2016–17, during the wet-dry transition, dry season, and dry-wet season (S1 Table in S1 File). The only exception to the simultaneous collections was during heavy rain and /or strong wind. Mosquitoes were collected with a manual vacuum aspirator. Two collectors worked each night inn each habitat, using HLC in the peridomestic area and ST in the forest fringe; each unit was sampled once. Variation in the number of collectors was due to their availability during field trips. Every hour, mosquitoes were euthanized with ethyl acetate (C_4_H_8_O_2_) vapors in the field and stored in silica gel identified by sampling unit number, habitat, date, and hour for subsequent species identification using the morphological identification key of Forattini [58]. The latitude and longitude of each collection point was recorded in the field with a GPS unit, using geodetic, global geographic coordinate WGS84.

The decision for collecting mosquitoes with ST at the forest fringe habitat considered the effectiveness of the trap for sampling Culicidae diversity [59]. These two collection methods have been compared previously in the Amazonian forest, and abundance and presence of *Ny*. *darlingi* were similar [60]. The sampling effort for each collection method and habitat was the same.

### Landscape metrics

Landscape metrics were generated from a Sentinel 2A satellite imagery (European Space Agency (ESA)) from the day nearest to the date of collection. with a spatial resolution of 10 m. Selection of the images included 1) the lowest percentage of clouds, and 2) as near as possible to the field collection date. The images were downloaded from the ESA (https://www.esa.int/ESA) in raster format and graded, with radiometric and atmospheric corrections, by means of an algorithm developed by ESA and implemented in Sen2Cor software [61]. The satellite images were classified in two land-use classes (forest and non-forest) through a supervised classification, using the maximum likelihood method. All analyzes were performed in QGIS, version 2.8 without any changes (QGIS is licensed under Creative Commons Attribution-ShareAlike 3.0 license (CC BY-SA) (https://creativecommons.org/licenses/by-sa/3.0/)).

Percentage of forest cover (PLAND) and forest edge density (ED) were calculated for all 79 sampling units (1 km radius). Details on how to calculate these metrics can be found in the S1 File [62]. PLAND and ED were also measured on a subtended circle of 0.5 km radius (0.8 km^2^), measured around each collection point (HLC/ST). These radii were chosen because in a study performed in Malaysian Borneo, Brock and collaborators [63] demonstrated that the optimal radius for addressing the edge effects on *P*. *knowlesi* malaria occurrence was 0.5 km, but the proportion of cleared land within 1 km was low.

PLAND and ED were chosen because of the ecological characteristics of *Ny*. *darlingi*, which have an affinity for partly shaded habitats for oviposition and development of immature stages in ground pools and slow-moving streams at forest edges, as reviewed by Hiwat and Bretas [34]. These metrics were used to assess the effects of landscape elements on mosquito abundance and: i) the forest cover (PLAND), ii) fragmentation (ED), and iii) the relationship between PLAND and ED (landscape structure).

ED measured at the 0.5 km radius was grouped into three categories—low (0–1.5 m / ha x 100); moderate (1.5–2.0 m / ha x 100) and high (2.0–3.5 m / ha x 100) and was always used in posterior analysis as a random effect and as categorical variables (low, moderate and high). These followed results from [56, 63], and the equal distribution of samples categories, with the mosquito collection point as the random effect. To define the ED categories as a random effect, we employed the results of the descriptive analysis that demonstrated differences in mosquito biodiversity in each specific ED category defined here.

PLAND and ED measured at every sampling unit (1km radius) were used both as continuous and categorical variables. The continuous was also used as a predictor, while the categorical entered as a random effect in the analysis. Combinations of PLAND and ED were classified in four categories of landscape (S2 Fig in S1 File) once we decided to test the hypothesis that modification in the landscape structure (herein considered to be a combination of PLAND and ED metrics in each sampling unit area) shifts in mosquito species composition and *Ny*. *darlingi* abundance. The categories were: A–PLAND ≤ 50% and ED < 0.015 m/ha; B–PLAND ≤ 50% and ED ≥ 0.015 m/ha; C–PLAND > 50% and ED ≥ 0.015 m/ha; D–PLAND > 50% and ED < 0.015 m/ha. The overview of the sample distribution according to landscape structure analysis (categories A, B, C, D) is presented in S3 Fig in S1 File.

We also measured the distance from each of the 79 sampling units to the nearest water drainage network (DW). Each network was constructed from the Digital Elevation Model (DEM) available on ESA. The spectral bands of each of the images were composed of bands 2 (Blue; 0.49 μm), 3 (Green; 0.56 μm) and 4 (Red; 0.66 μm) to generate a final-colored image for each region of the collection points. The drainage network indicates where water is most likely to accumulate according to the terrain relief but does not necessarily indicate locations of rivers or streams.

### Diversity indices

The diversity indices of Margalef, Shannon, Simpson and Berger-Parker for each sampling unit were calculated by the Rényi series, based on Hill’s measures [64]. As diversity indexes are highly autocorrelated, we used the Shannon index in analytical associations because it has links to entropy theory [65].

### Statistical analysis

Data analyses were conducted in three steps: 1) descriptive statistics, and 2) analytical associations. To construct the models, and prior to commencing the analytical approach, we applied extensive descriptive statistics. We assessed the sampling unit distribution according to the landscape structure and vegetation class (S3 Table in S1 File). The units that corresponded to landscape D and Open Tropical Moist Forest (OTMF) showed the lowest mean *Ny*. *darlingi* abundance and were considered the baseline group to create dummy variables for generalized linear mixed model (GLMM) and regression analysis.

For step 1, Spearman’s correlation index (at a significance level of 5%) was utilized to verify the correlation among predictor variables (diversity index, PLAND, ED, drainage network (DW), landscape structure—landscape categories A, B, C, D—vegetation class), and number of *Ny*. *darlingi*. Principal Component Analysis (PCA) was used in exploratory analyses to identify associations among the number of *Ny*. *darlingi*, PLAND, ED and Shannon index variation according to the four categories of landscape structure. The non-parametric Kolmogorov-Smirnov test was employed to verify whether the variables had a normal distribution (at a significance level of 5%). To compare the distribution of the *Ny*. *darlingi* abundance among the different landscape categories and five vegetation classes found in our field collection areas in the Amazon: Anthropogenic (Atp), Open Tropical Moist Forest (OTMF), Open Tropical Moist Forest with Arecaceae dominance (OTMFA), Tropical Moist Forest (TMF) and Campinarana (Cpn) (Brazilian Institute of Geography and Statistics—IBGE classification) [55], we applied the Kruskal-Wallis with Mann-Whitney-Wilcoxon’s multiple comparison test with Bonferroni correction. The analysis of variance (oneway ANOVA) with Tukey’s corrections was applied to verify Shannon-index variation among landscapes categories, and to test the null hypothesis that landscape structure does not influence species abundance, considering 5% as the significance level. Tukey’s correction was employed to compare the landscape structure categories in five classes of vegetation found in our collection localities. Variation in species composition among sampling units in each landscape structure category was addressed employing Canonical correspondence analysis -CCA in R-project, vegan package [66].

In the step 2, we used a simple linear regression and generalized linear mixed models (GLMM) to verify the relationship between *Ny*. *darlingi* abundance and the Shannon index, PLAND, ED and DW. We employed the GLMM statistical analyses because both edge density and landscape categories were random variables. A table with all models can be found in S2 Table in S1 File. In all models we employed PLAND and ED measured at each sampling unit (3.14 km^2^), as predictor variables, while ED measured at 0.5 km radius (both from peridomestic and forest fringe habitats) and classified as categorical variables, was used as a random effect. The two mosquito sampling points (ST and HLC) and their mosquito diversity assemblages were assessed together as fixed variables. Four simple regression models were constructed—Shannon index, PLAND, ED and DW. For GLMM, three models were constructed, all of them using Shannon index, PLAND, and DW as fixed effects, and ED categories (low, moderate, high) at peridomestic or forest fringe habitat, or landscape structure categories as random effects. The PLAND and ED metrics were calculated at the 1 km radius and used to categorize landscapes A–D only. For the models (1) and (2), the ED was calculated at the 0.5 km radius for both peridomestic and forest fringe habitats and used as a random effect. The center of the 0.5 km circle of both peridomestic and forest fringe habitats were inside a 1 km radius circle. Mosquito data from peridomestic and forest fringe habitats were combined for the GLMM analysis. The choice of using ED as a random effect were based on the results of the descriptive analysis, which showed a statistically significant difference in Shannon-index (S3 Fig in S1 File) to Culicidae biodiversity. The categorical approach to PLAND analysis did not show differences for Shannon-index variance (*P* > 0.05).

We used path analysis to test the prediction that changes in *Ny*. *darlingi* abundance with increasing Shannon index are the cause of changes in richness (number of different taxonomic mosquito species morphologically and/or molecularly distinct) [58, 64, 67, 68], and to quantify the direct effects on *Ny*. *darlingi* abundance caused by variation in the Shannon index, PLAND, ED, landscape structure categories (as dummy variable), vegetation classes (as dummy variable) and DW, while removing the effects of other independent variables [69, 70]. We inserted the vegetation class to verify whether it represents a confusion variable [71]. We employed the Structural Equation Model (SEM) to visualize the model using a path diagram with “semPlot” package of R-project v. 3.6.

The GLMM models were adjusted by the distance between the sampling units and drainage networks because of the bionomic characteristics of *Ny*. *darlingi* as an ecotone species that lay eggs in partially shaded habitats, including forest fringe [20, 34]. All statistical analysis was carried out using RStudio v.1.1.456 software (R Development Core Team, R Foundation for Statistical Computing, Austria) R-project (available at http://www.r-project.org), and “BiodiversityR”, “wordcloud” and “nlme” packages.

## Results

### Mosquito collection

In total, 25,323 specimens of 137 species in 17 genera were collected using the HLC and Shannon trap. *Nyssorhynchus darlingi* corresponded to 46.6% (11,810 specimens) of this total. Overall, the most abundant species were those in the subfamily Anophelinae, 56.4% (14,281), with *Ny*. *darlingi* comprising 82.7% (11,810) of the total Anophelinae collections in the peridomestic habitat (Table 1). The next most abundant groups were the Mansoniini tribe, comprising 19.4% (4,917), Culicini 18.7% (4,743) and Aedini 3.8% (950). Mosquitoes of the Aedeomyiini, Uranotaeniini and Sabethini tribes comprised 1.2% of the total collected (S4 Fig in S1 File).

**Table 1:** Species list of Culicidae collected in malaria endemic areas in Brazilian Amazon.

			PLAND (%)																				Edge density (m / ha)											
			Peridomestic (HLC)										Forest fringe (Shannon trap)										Peridomestic (HLC)						Forest fringe (Shannon trap)					
			≤ 25		25 ¬ 45		45 ¬ 60		60 ¬ 75		> 75		≤ 25		25 ¬ 45		45 ¬ 60		60 ¬ 75		> 75		0 ¬ 1.5		1.5 ¬ 2.0		2.0 ¬ 3.5		0 ¬ 1.5		1.5 ¬ 2.0		2.0 ¬ 3.5	
**Species**	**Author**	**Taxonomic group**	**n**	**n/%**	**n**	**n/%**	**n**	**n/%**	**n**	**n/%**	**n**	**n/%**	**n**	**n/%**	**n**	**n/%**	**n**	**n/%**	**n**	**n/%**	**n**	**n/%**	**n**	**n/%**	**n**	**n/%**	**n**	**n/%**	**n**	**n/%**	**n**	**n/%**	**n**	**n/%**
*Aedeomyia squamipennis*	(Lynch Arribalzaga)	Aedeomyiini	3	0.1	0	0.0	6	0.2	1	0.0	2	0.1	136	4.1	60	2.6	81	4.1	4	0.2	9	0.5	2	0.1	3	0.1	7	0.2	135	4.6	62	1.7	93	1.7
*Aedes albopictus*	(Skuse)	Aedini	0	0.0	0	0.0	0	0.0	0	0.0	0	0.0	1	0.0	0	0.0	0	0.0	0	0.0	0	0.0	0	0.0	0	0.0	0	0.0	1	0.0	0	0.0	0	0.0
*Aedes argyrothorax*	Bonne-Wepster and Bonne	Aedini	0	0.0	0	0.0	0	0.0	0	0.0	1	0.1	0	0.0	0	0.0	0	0.0	2	0.1	0	0.0	0	0.0	0	0.0	1	0.0	1	0.0	0	0.0	1	0.0
*Aedes fulvithorax*	(Lutz)	Aedini	0	0.0	1	0.0	0	0.0	0	0.0	0	0.0	0	0.0	0	0.0	0	0.0	0	0.0	0	0.0	0	0.0	1	0.0	0	0.0	0	0.0	0	0.0	0	0.0
*Aedes fulvus*	(Wiedemann)	Aedini	0	0.0	0	0.0	0	0.0	0	0.0	0	0.0	45	1.3	11	0.5	5	0.3	20	0.8	58	3.4	0	0.0	0	0.0	0	0.0	53	1.8	57	1.6	29	0.5
*Aedes fulvus sp1*		Aedini	0	0.0	0	0.0	0	0.0	0	0.0	0	0.0	7	0.2	0	0.0	0	0.0	3	0.1	0	0.0	0	0.0	0	0.0	0	0.0	7	0.2	0	0.0	3	0.1
*Aedes hastatus*	Dyar	Aedini	0	0.0	0	0.0	0	0.0	0	0.0	0	0.0	0	0.0	0	0.0	1	0.1	0	0.0	0	0.0	0	0.0	0	0.0	0	0.0	0	0.0	0	0.0	1	0.0
*Aedes hortator*	Dyar and Knab	Aedini	0	0.0	0	0.0	0	0.0	0	0.0	0	0.0	0	0.0	0	0.0	0	0.0	3	0.1	0	0.0	0	0.0	0	0.0	0	0.0	0	0.0	0	0.0	3	0.1
*Aedes infirmatus*	Dyar and Knab	Aedini	0	0.0	0	0.0	0	0.0	0	0.0	0	0.0	0	0.0	0	0.0	2	0.1	0	0.0	0	0.0	0	0.0	0	0.0	0	0.0	0	0.0	0	0.0	2	0.0
*Aedes scapularis*	(Rondani)	Aedini	0	0.0	0	0.0	0	0.0	1	0.0	2	0.1	1	0.0	5	0.2	0	0.0	10	0.4	13	0.8	1	0.0	2	0.0	0	0.0	15	0.5	14	0.4	0	0.0
*Aedes serratus*	(Theobald)	Aedini	0	0.0	0	0.0	1	0.0	0	0.0	2	0.1	19	0.6	8	0.3	1	0.1	46	1.7	12	0.7	0	0.0	0	0.0	3	0.1	31	1.0	13	0.4	41	0.8
*Aedes serratus / nubilus*		Aedini	0	0.0	0	0.0	0	0.0	0	0.0	0	0.0	16	0.5	5	0.2	1	0.1	32	1.2	18	1.1	0	0.0	0	0.0	0	0.0	15	0.5	26	0.7	31	0.6
*Psorophora albigenu*	(Peryassú)	Aedini	0	0.0	0	0.0	0	0.0	1	0.0	0	0.0	2	0.1	2	0.1	7	0.4	21	0.8	10	0.6	0	0.0	1	0.0	0	0.0	14	0.5	15	0.4	13	0.2
*Psorophora albipes*	(Theobald)	Aedini	0	0.0	0	0.0	0	0.0	5	0.2	0	0.0	4	0.1	19	0.8	36	1.8	32	1.2	1	0.1	0	0.0	5	0.1	0	0.0	25	0.8	27	0.8	40	0.7
*Psorophora amazonensis*	(Theobald)	Aedini	0	0.0	0	0.0	0	0.0	0	0.0	0	0.0	0	0.0	0	0.0	0	0.0	2	0.1	0	0.0	0	0.0	0	0.0	0	0.0	0	0.0	1	0.0	1	0.0
*Psorophora cingulata*	(Fabricius)	Aedini	1	0.0	6	0.2	23	0.9	19	0.8	21	1.2	65	1.9	42	1.8	173	8.7	47	1.8	32	1.9	3	0.1	26	0.4	45	1.0	61	2.1	42	1.2	248	4.5
*Psorophora dimidiata*	Cerqueira	Aedini	1	0.0	0	0.0	0	0.0	1	0.0	0	0.0	0	0.0	1	0.0	2	0.1	1	0.0	0	0.0	0	0.0	1	0.0	1	0.0	2	0.1	2	0.1	0	0.0
*Psorophora discrucians*	(Walker)	Aedini	0	0.0	1	0.0	0	0.0	0	0.0	0	0.0	0	0.0	0	0.0	0	0.0	0	0.0	0	0.0	0	0.0	1	0.0	0	0.0	0	0.0	0	0.0	0	0.0
*Psorophora ferox*	(von Humboldt)	Aedini	0	0.0	0	0.0	0	0.0	0	0.0	0	0.0	4	0.1	1	0.0	2	0.1	4	0.2	3	0.2	0	0.0	0	0.0	0	0.0	3	0.1	4	0.1	7	0.1
*Psorophora saeva*	Dyar and Knab	Aedini	0	0.0	0	0.0	0	0.0	0	0.0	0	0.0	4	0.1	0	0.0	0	0.0	0	0.0	1	0.1	0	0.0	0	0.0	0	0.0	4	0.1	1	0.0	0	0.0
*Anopheles costai*	da Fonseca & da Silva Ramos	Anophelinae	0	0.0	0	0.0	0	0.0	0	0.0	0	0.0	0	0.0	4	0.2	0	0.0	18	0.7	17	1.0	0	0.0	0	0.0	0	0.0	4	0.1	3	0.1	32	0.6
*Anopheles costai* (near)		Anophelinae	0	0.0	0	0.0	0	0.0	0	0.0	0	0.0	0	0.0	4	0.2	0	0.0	0	0.0	0	0.0	0	0.0	0	0.0	0	0.0	1	0.0	3	0.1	0	0.0
*Anopheles costai* G1		Anophelinae	0	0.0	0	0.0	0	0.0	0	0.0	2	0.1	0	0.0	0	0.0	5	0.3	2	0.1	6	0.4	0	0.0	0	0.0	2	0.1	1	0.0	5	0.1	7	0.1
*Anopheles costai* G2		Anophelinae	0	0.0	0	0.0	0	0.0	0	0.0	3	0.2	0	0.0	0	0.0	0	0.0	0	0.0	0	0.0	0	0.0	0	0.0	3	0.1	0	0.0	0	0.0	0	0.0
*Anopheles costai* G3		Anophelinae	0	0.0	0	0.0	0	0.0	0	0.0	0	0.0	0	0.0	11	0.5	1	0.1	0	0.0	5	0.3	0	0.0	0	0.0	0	0.0	7	0.2	10	0.3	0	0.0
*Anopheles costai* G4		Anophelinae	0	0.0	0	0.0	0	0.0	0	0.0	1	0.1	0	0.0	0	0.0	0	0.0	0	0.0	4	0.2	0	0.0	0	0.0	1	0.0	0	0.0	0	0.0	4	0.1
*Anopheles costai* G5		Anophelinae	0	0.0	0	0.0	0	0.0	0	0.0	0	0.0	0	0.0	0	0.0	0	0.0	0	0.0	1	0.1	0	0.0	0	0.0	0	0.0	0	0.0	1	0.0	0	0.0
*Anopheles fluminensis* (*latu sensu*)		Anophelinae	0	0.0	0	0.0	0	0.0	0	0.0	0	0.0	0	0.0	0	0.0	0	0.0	4	0.2	0	0.0	0	0.0	0	0.0	0	0.0	0	0.0	2	0.1	2	0.0
*Anopheles fluminensis* (*strictu sensu*)	Root	Anophelinae	0	0.0	0	0.0	0	0.0	0	0.0	0	0.0	0	0.0	0	0.0	0	0.0	0	0.0	5	0.3	0	0.0	0	0.0	0	0.0	0	0.0	5	0.1	0	0.0
*Anopheles fluminensis G1*		Anophelinae	0	0.0	0	0.0	0	0.0	0	0.0	0	0.0	0	0.0	0	0.0	0	0.0	1	0.0	4	0.2	0	0.0	0	0.0	0	0.0	1	0.0	4	0.1	0	0.0
*Anopheles fluminensis* G2		Anophelinae	0	0.0	0	0.0	0	0.0	0	0.0	0	0.0	0	0.0	0	0.0	0	0.0	0	0.0	5	0.3	0	0.0	0	0.0	0	0.0	0	0.0	5	0.1	0	0.0
*Anopheles fluminensis* (near)		Anophelinae	0	0.0	0	0.0	0	0.0	0	0.0	0	0.0	0	0.0	0	0.0	0	0.0	0	0.0	2	0.1	0	0.0	0	0.0	0	0.0	0	0.0	2	0.1	0	0.0
*Anopheles forattini*	Wilkerson and Sallum	Anophelinae	0	0.0	0	0.0	0	0.0	0	0.0	0	0.0	7	0.2	0	0.0	1	0.1	0	0.0	0	0.0	0	0.0	0	0.0	0	0.0	8	0.3	0	0.0	0	0.0
*Anopheles malefactor* (near)	Dyar and Knab	Anophelinae	0	0.0	0	0.0	0	0.0	0	0.0	0	0.0	0	0.0	0	0.0	0	0.0	0	0.0	1	0.1	0	0.0	0	0.0	0	0.0	0	0.0	1	0.0	0	0.0
*Anopheles mattogrossensis*	Lutz and Neiva	Anophelinae	0	0.0	0	0.0	0	0.0	5	0.2	0	0.0	0	0.0	1	0.0	0	0.0	2	0.1	0	0.0	0	0.0	5	0.1	0	0.0	2	0.1	0	0.0	1	0.0
*Anopheles minor*	Da Costa Lima	Anophelinae	0	0.0	0	0.0	0	0.0	0	0.0	0	0.0	0	0.0	2	0.1	7	0.4	0	0.0	0	0.0	0	0.0	0	0.0	0	0.0	8	0.3	1	0.0	0	0.0
*Anopheles peryassui*	Dyar and Knab	Anophelinae	0	0.0	1	0.0	0	0.0	1	0.0	0	0.0	0	0.0	1	0.0	20	1.0	25	1.0	20	1.2	0	0.0	0	0.0	2	0.1	0	0.0	32	0.9	34	0.6
*Anopheles punctimacula*	Dyar and Knab	Anophelinae	0	0.0	0	0.0	0	0.0	0	0.0	4	0.2	0	0.0	0	0.0	0	0.0	0	0.0	11	0.7	0	0.0	0	0.0	4	0.1	0	0.0	0	0.0	11	0.2
*Anopheles punctimacula* (near)		Anophelinae	0	0.0	0	0.0	0	0.0	0	0.0	0	0.0	0	0.0	0	0.0	0	0.0	0	0.0	1	0.1	0	0.0	0	0.0	0	0.0	0	0.0	0	0.0	1	0.0
*Anopheles rangeli*	Gabaldon, Cova García and Lopez	Anophelinae	5	0.1	0	0.0	0	0.0	35	1.5	5	0.3	4	0.1	1	0.0	3	0.2	18	0.7	19	1.1	0	0.0	44	0.7	1	0.0	20	0.7	23	0.6	2	0.0
*Anopheles shannoni*	Davis	Anophelinae	0	0.0	0	0.0	0	0.0	0	0.0	0	0.0	1	0.0	0	0.0	0	0.0	0	0.0	0	0.0	0	0.0	0	0.0	0	0.0	1	0.0	0	0.0	0	0.0
*Anopheles sp*.		Anophelinae	0	0.0	0	0.0	0	0.0	0	0.0	0	0.0	1	0.0	0	0.0	0	0.0	1	0.0	0	0.0	0	0.0	0	0.0	0	0.0	0	0.0	1	0.0	1	0.0
*Anopheles squamifemur*	Antunes	Anophelinae	0	0.0	0	0.0	0	0.0	0	0.0	0	0.0	0	0.0	1	0.0	0	0.0	2	0.1	0	0.0	0	0.0	0	0.0	0	0.0	1	0.0	0	0.0	2	0.0
*Chagasia fajardi*	(Lutz)	Anophelinae	0	0.0	1	0.0	0	0.0	0	0.0	0	0.0	0	0.0	5	0.2	1	0.1	0	0.0	2	0.1	1	0.0	0	0.0	0	0.0	7	0.2	1	0.0	0	0.0
*Chagasia sp*.		Anophelinae	0	0.0	0	0.0	0	0.0	0	0.0	0	0.0	0	0.0	0	0.0	0	0.0	3	0.1	0	0.0	0	0.0	0	0.0	0	0.0	0	0.0	0	0.0	3	0.1
*Kerteszia neivai*	Howard, Dyar and Knab	Anophelinae	0	0.0	0	0.0	0	0.0	0	0.0	0	0.0	0	0.0	0	0.0	0	0.0	2	0.1	2	0.1	0	0.0	0	0.0	0	0.0	2	0.1	2	0.1	0	0.0
*Nyssorhynchus albitarsis*	Lynch Arribálzaga	Anophelinae	3	0.1	1	0.0	3	0.1	5	0.2	0	0.0	1	0.0	0	0.0	0	0.0	0	0.0	0	0.0	3	0.1	5	0.1	4	0.1	1	0.0	0	0.0	0	0.0
*Nyssorhynchus albitarsis* H		Anophelinae	1	0.0	2	0.1	0	0.0	0	0.0	0	0.0	0	0.0	0	0.0	0	0.0	0	0.0	0	0.0	1	0.0	2	0.0	0	0.0	0	0.0	0	0.0	0	0.0
*Nyssorhynchus arthuri* C		Anophelinae	0	0.0	2	0.1	0	0.0	0	0.0	0	0.0	0	0.0	0	0.0	0	0.0	12	0.5	0	0.0	2	0.1	0	0.0	0	0.0	0	0.0	0	0.0	12	0.2
*Nyssorhynchus benarrochi*	Gabaldon, Cova-García and Lopez	Anophelinae	6		0		0		0		0		0		1		0		0		0		0		0		6		0		1		0	0.0
*Nyssorhynchus benarrochi* B		Anophelinae	0	0.0	2	0.1	0	0.0	0	0.0	1	0.1	0	0.0	0	0.0	0	0.0	0	0.0	0	0.0	0	0.0	2	0.0	1	0.0	0	0.0	0	0.0	0	0.0
*Nyssorhynchus benarrochi* G1		Anophelinae	0	0.0	0	0.0	0	0.0	0	0.0	0	0.0	3	0.1	0	0.0	0	0.0	0	0.0	0	0.0	0	0.0	0	0.0	0	0.0	3	0.1	0	0.0	0	0.0
*Nyssorhynchus benarrochi* G2		Anophelinae	0	0.0	1	0.0	0	0.0	0	0.0	0	0.0	0	0.0	1	0.0	1	0.1	0	0.0	0	0.0	1	0.0	0	0.0	0	0.0	1	0.0	1	0.0	0	0.0
*Nyssorhynchus braziliensis*	(Chagas)	Anophelinae	59	1.5	8	0.3	3	0.1	1	0.0	0	0.0	3	0.1	4	0.2	66	3.3	3	0.1	0	0.0	3	0.1	1	0.0	67	1.5	3	0.1	73	2.0	0	0.0
*Nyssorhynchus darlingi*		Anophelinae	3473	86.9	2082	82.2	1839	71.0	1835	77.1	1291	70.8	395	11.8	285	12.2	179	9.0	60	2.3	371	21.9	2143	82.0	5493	87.0	2884	65.4	545	18.4	363	10.1	414	7.6
*Nyssorhynchus deaneorum*	Rosa-Freitas	Anophelinae	64	1.6	0	0.0	0	0.0	11	0.5	0	0.0	7	0.2	0	0.0	1	0.1	1	0.0	0	0.0	0	0.0	75	1.2	0	0.0	6	0.2	3	0.1	0	0.0
*Nyssorhynchus dunhami*	Causey	Anophelinae	0	0.0	0	0.0	0	0.0	0	0.0	0	0.0	0	0.0	1	0.0	1	0.1	0	0.0	0	0.0	0	0.0	0	0.0	0	0.0	0	0.0	1	0.0	1	0.0
*Nyssorhynchus galvaoi*	Causey, Deane and Deane	Anophelinae	0	0.0	0	0.0	0	0.0	10	0.4	0	0.0	0	0.0	0	0.0	0	0.0	0	0.0	0	0.0	10	0.4	0	0.0	0	0.0	0	0.0	0	0.0	0	0.0
*Nyssorhynchus goeldii*	Rozeboom and Gabaldon	Anophelinae	0	0.0	0	0.0	1	0.0	25	1.1	0	0.0	166	5.0	20	0.9	1	0.1	4	0.2	0	0.0	0	0.0	26	0.4	0	0.0	190	6.4	0	0.0	1	0.0
*Nyssorhynchus konderi*	Galvão & Damasceno	Anophelinae	4	0.1	0	0.0	0	0.0	1	0.0	8	0.4	5	0.2	1	0.0	1	0.1	0	0.0	100	5.9	8	0.3	0	0.0	5	0.1	5	0.2	101	2.8	1	0.0
*Nyssorhynchus konderi / oswaldoi*		Anophelinae	0	0.0	0	0.0	0	0.0	0	0.0	0	0.0	0	0.0	0	0.0	0	0.0	0	0.0	2	0.1	0	0.0	0	0.0	0	0.0	0	0.0	0	0.0	2	0.0
*Nyssorhynchus konderi* A		Anophelinae	0	0.0	0	0.0	0	0.0	0	0.0	1	0.1	0	0.0	0	0.0	0	0.0	0	0.0	61	3.6	1	0.0	0	0.0	0	0.0	0	0.0	61	1.7	0	0.0
*Nyssorhynchus konderi* B		Anophelinae	0	0.0	0	0.0	0	0.0	0	0.0	40	2.2	0	0.0	0	0.0	0	0.0	59	2.2	296	17.5	38	1.5	0	0.0	2	0.1	59	2.0	289	8.0	7	0.1
*Nyssorhynchus nuneztovari*		Anophelinae	1	0.0	0	0.0	0	0.0	1	0.0	0	0.0	0	0.0	0	0.0	0	0.0	1	0.0	0	0.0	0	0.0	0	0.0	2	0.1	0	0.0	0	0.0	1	0.0
*Nyssorhynchus nuneztovari* A		Anophelinae	1	0.0	0	0.0	0	0.0	12	0.5	0	0.0	0	0.0	0	0.0	0	0.0	0	0.0	0	0.0	0	0.0	12	0.2	1	0.0	0	0.0	0	0.0	0	0.0
*Nyssorhynchus oryzalimnetes*	Wilkerson and Motoki	Anophelinae	98	2.5	0	0.0	0	0.0	5	0.2	0	0.0	14	0.4	0	0.0	0	0.0	1	0.0	0	0.0	0	0.0	103	1.6	0	0.0	1	0.0	14	0.4	0	0.0
*Nyssorhynchus oswaldoi* (*latu sensu*)		Anophelinae	0	0.0	0	0.0	0	0.0	0	0.0	0	0.0	0	0.0	3	0.1	18	0.9	11	0.4	135	8.0	0	0.0	0	0.0	0	0.0	6	0.2	158	4.4	3	0.1
*Nyssorhynchus oswaldoi (strictu sensu)*	(Peryassú)	Anophelinae	0	0.0	0	0.0	1	0.0	0	0.0	0	0.0	0	0.0	0	0.0	0	0.0	0	0.0	0	0.0	1	0.0	0	0.0	0	0.0	0	0.0	0	0.0	0	0.0
*Nyssorhynchus oswaldoi* A		Anophelinae	0	0.0	0	0.0	0	0.0	0	0.0	0	0.0	0	0.0	15	0.6	5	0.3	2	0.1	1	0.1	0	0.0	0	0.0	0	0.0	9	0.3	9	0.3	5	0.1
*Nyssorhynchus sp*.		Anophelinae	0	0.0	0	0.0	0	0.0	1	0.0	0	0.0	0	0.0	0	0.0	0	0.0	0	0.0	0	0.0	0	0.0	0	0.0	1	0.0	0	0.0	0	0.0	0	0.0
*Nyssorhynchus triannulatus*	(Neiva and Pinto)	Anophelinae	0	0.0	27	1.1	7	0.3	22	0.9	3	0.2	136	4.1	210	9.0	132	6.7	174	6.6	21	1.2	42	1.6	14	0.2	3	0.1	391	13.2	103	2.9	179	3.3
*Stethomyia thomasi*	Shannon	Anophelinae	0	0.0	0	0.0	0	0.0	0	0.0	0	0.0	0	0.0	0	0.0	0	0.0	1	0.0	0	0.0	0	0.0	0	0.0	0	0.0	1	0.0	0	0.0	0	0.0
*Culex* (*Aedinus*) *amazonensis*	(Lutz)	Culicini	0	0.0	0	0.0	0	0.0	0	0.0	0	0.0	0	0.0	0	0.0	0	0.0	30	1.1	0	0.0	0	0.0	0	0.0	0	0.0	0	0.0	0	0.0	30	0.6
*Culex* (*Anoedioporpa*) *originator*	Gordon and Evans	Culicini	0	0.0	0	0.0	0	0.0	0	0.0	0	0.0	0	0.0	0	0.0	0	0.0	0	0.0	1	0.1	0	0.0	0	0.0	0	0.0	1	0.0	0	0.0	0	0.0
*Culex* (*Anoedioporpa*) *sp*.		Culicini	0	0.0	0	0.0	0	0.0	0	0.0	0	0.0	1	0.0	1	0.0	1	0.1	0	0.0	0	0.0	0	0.0	0	0.0	0	0.0	2	0.1	0	0.0	1	0.0
*Culex* (*Culex*) *declarator*	Dyar and Knab	Culicini	0	0.0	0	0.0	6	0.2	1	0.0	8	0.4	26	0.8	127	5.5	31	1.6	28	1.1	2	0.1	6	0.2	4	0.1	5	0.1	53	1.8	64	1.8	99	1.8
*Culex* (*Culex*) Grupo Coronator		Culicini	1	0.0	2	0.1	2	0.1	0	0.0	3	0.2	15	0.5	30	1.3	17	0.9	17	0.6	20	1.2	1	0.0	5	0.1	2	0.1	42	1.4	12	0.3	45	0.8
*Culex* (*Culex*) *mollis*	Dyar and Knab	Culicini	1	0.0	0	0.0	5	0.2	2	0.1	9	0.5	1	0.0	252	10.8	8	0.4	7	0.3	36	2.1	7	0.3	4	0.1	6	0.1	72	2.4	214	5.9	17	0.3
*Culex* (*Culex*) *nigripalpus*	Theobald	Culicini	0	0.0	0	0.0	0	0.0	0	0.0	0	0.0	0	0.0	2	0.1	2	0.1	4	0.2	0	0.0	0	0.0	0	0.0	0	0.0	2	0.1	1	0.0	5	0.1
*Culex* (*Culex*) *quinquefasciatus*	Say	Culicini	171	4.3	153	6.0	98	3.8	126	5.3	15	0.8	0	0.0	0	0.0	5	0.3	0	0.0	10	0.6	98	3.8	36	0.6	429	9.7	1	0.0	7	0.2	11	0.2
*Culex* (*Culex*) *sp*.		Culicini	0	0.0	0	0.0	2	0.1	0	0.0	0	0.0	6	0.2	24	1.0	212	10.7	34	1.3	5	0.3	0	0.0	0	0.0	2	0.1	34	1.2	206	5.7	42	0.8
*Culex* (*Melanoconion*) *adamesi*	Sirivanakarn and Galindo	Culicini	0	0.0	0	0.0	0	0.0	0	0.0	0	0.0	0	0.0	0	0.0	7	0.4	24	0.9	2	0.1	0	0.0	0	0.0	0	0.0	0	0.0	3	0.1	35	0.6
*Culex* (*Melanoconion*) *aureonotatus*	Duret and Barreto	Culicini	0	0.0	0	0.0	0	0.0	0	0.0	0	0.0	1	0.0	5	0.2	0	0.0	3	0.1	0	0.0	0	0.0	0	0.0	0	0.0	2	0.1	0	0.0	7	0.1
*Culex* (*Melanoconion*) *bastagarius*	Dyar and Knab	Culicini	19	0.5	14	0.6	4	0.2	1	0.0	134	7.4	100	3.0	99	4.3	54	2.7	130	4.9	130	7.7	117	4.5	33	0.5	22	0.5	162	5.5	164	4.6	187	3.4
*Culex* (*Melanoconion*) *clarki*	Evans	Culicini	0	0.0	0	0.0	1	0.0	0	0.0	0	0.0	0	0.0	0	0.0	1	0.1	0	0.0	1	0.1	0	0.0	0	0.0	1	0.0	0	0.0	0	0.0	1	0.0
*Culex* (*Melanoconion*) *crybda*	Dyar	Culicini	0	0.0	0	0.0	1	0.0	1	0.0	0	0.0	0	0.0	0	0.0	8	0.4	9	0.3	1	0.1	0	0.0	1	0.0	1	0.0	2	0.1	5	0.1	10	0.2
*Culex* (*Melanoconion*) *crybda sp1*		Culicini	0	0.0	0	0.0	0	0.0	0	0.0	0	0.0	3	0.1	64	2.8	1	0.1	29	1.1	11	0.7	0	0.0	0	0.0	0	0.0	27	0.9	8	0.2	73	1.3
*Culex* (*Melanoconion*) *eknomios*	Forattini and Sallum	Culicini	0	0.0	1	0.0	0	0.0	0	0.0	0	0.0	0	0.0	2	0.1	3	0.2	1	0.0	2	0.1	0	0.0	1	0.0	0	0.0	4	0.1	2	0.1	2	0.0
*Culex* (*Melanoconion*) *ensiformis*	Bonne-Wepster and Bonne	Culicini	0	0.0	0	0.0	0	0.0	0	0.0	0	0.0	6	0.2	0	0.0	1	0.1	2	0.1	1	0.1	0	0.0	0	0.0	0	0.0	6	0.2	0	0.0	4	0.1
*Culex* (*Melanoconion*) *gnomatos*	Sallum, Hutchings, Leila and Ferreira	Culicini	4	0.1	27	1.1	0	0.0	1	0.0	0	0.0	43	1.3	90	3.9	6	0.3	94	3.6	1	0.1	0	0.0	0	0.0	32	0.7	5	0.2	91	2.5	138	2.5
*Culex* (*Melanoconion*) Grupo Atratus		Culicini	0	0.0	0	0.0	0	0.0	0	0.0	0	0.0	19	0.6	11	0.5	27	1.4	1	0.0	6	0.4	0	0.0	0	0.0	0	0.0	32	1.1	31	0.9	1	0.0
*Culex* (*Melanoconion*) Grupo Educator		Culicini	0	0.0	2	0.1	0	0.0	0	0.0	0	0.0	0	0.0	7	0.3	0	0.0	0	0.0	0	0.0	0	0.0	0	0.0	2	0.1	0	0.0	7	0.2	0	0.0
*Culex* (*Melanoconion*) Grupo Pilosus		Culicini	0	0.0	0	0.0	0	0.0	0	0.0	1	0.1	0	0.0	2	0.1	1	0.1	5	0.2	3	0.2	0	0.0	1	0.0	0	0.0	5	0.2	2	0.1	4	0.1
*Culex* (*Melanoconion*) *inadmirabilis*	Dyar	Culicini	0	0.0	0	0.0	0	0.0	0	0.0	0	0.0	0	0.0	24	1.0	0	0.0	0	0.0	0	0.0	0	0.0	0	0.0	0	0.0	0	0.0	24	0.7	0	0.0
*Culex* (*Melanoconion*) *ocossa*	Dyar and Knab	Culicini	0	0.0	0	0.0	3	0.1	0	0.0	12	0.7	0	0.0	0	0.0	12	0.6	1	0.0	0	0.0	0	0.0	12	0.2	3	0.1	1	0.0	12	0.3	0	0.0
*Culex* (*Melanoconion*) *pedroi*	Sirivanakarn and Belkin	Culicini	0	0.0	0	0.0	0	0.0	0	0.0	0	0.0	27	0.8	1	0.0	0	0.0	16	0.6	0	0.0	0	0.0	0	0.0	0	0.0	13	0.4	1	0.0	30	0.6
*Culex* (*Melanoconion*) *portesi*	Senevet and Abonnenc	Culicini	3	0.1	48	1.9	1	0.0	2	0.1	11	0.6	335	10.0	352	15.1	40	2.0	175	6.6	6	0.4	1	0.0	2	0.0	62	1.4	0	0.0	392	10.9	516	9.4
*Culex* (*Melanoconion*) *ribeirensis*	Forattini and Sallum	Culicini	0	0.0	0	0.0	0	0.0	0	0.0	0	0.0	0	0.0	2	0.1	0	0.0	6	0.2	0	0.0	0	0.0	0	0.0	0	0.0	2	0.1	5	0.1	1	0.0
*Culex* (*Melanoconion*) *sp*.		Culicini	1	0.0	14	0.6	3	0.1	0	0.0	2	0.1	26	0.8	146	6.3	44	2.2	163	6.2	50	3.0	6	0.2	3	0.1	11	0.3	51	1.7	126	3.5	252	4.6
*Culex* (*Melanoconion*) *sp*. Seção Melanoconion		Culicini	5	0.1	10	0.4	0	0.0	1	0.0	0	0.0	0	0.0	2	0.1	3	0.2	6	0.2	0	0.0	0	0.0	2	0.0	14	0.3	2	0.1	3	0.1	6	0.1
*Culex* (*Melanoconion*) *spissipes*	(Theobald)	Culicini	0	0.0	0	0.0	0	0.0	0	0.0	0	0.0	1	0.0	0	0.0	0	0.0	13	0.5	1	0.1	0	0.0	0	0.0	0	0.0	1	0.0	1	0.0	13	0.2
*Culex* (*Melanoconion*) Seção Spissipes		Culicini	0	0.0	0	0.0	0	0.0	0	0.0	0	0.0	0	0.0	2	0.1	2	0.1	0	0.0	0	0.0	0	0.0	0	0.0	0	0.0	2	0.1	2	0.1	0	0.0
*Culex* (*Melanoconion*) *theobaldi*	(Lutz)	Culicini	10	0.3	31	1.2	6	0.2	30	1.3	63	3.5	2	0.1	1	0.0	13	0.7	15	0.6	30	1.8	7	0.3	81	1.3	52	1.2	22	0.7	18	0.5	21	0.4
*Culex* (*Melanoconion*) *vaxus*	Dyar	Culicini	0	0.0	41	1.6	0	0.0	6	0.3	0	0.0	1	0.0	7	0.3	0	0.0	0	0.0	1	0.1	2	0.1	45	0.7	0	0.0	3	0.1	3	0.1	3	0.1
*Culex* (*Melanoconion*) *vomerifer*	Komp	Culicini	0	0.0	6	0.2	0	0.0	0	0.0	0	0.0	11	0.3	17	0.7	18	0.9	28	1.1	2	0.1	0	0.0	0	0.0	6	0.1	0	0.0	13	0.4	63	1.2
*Culex* (*Melanoconion*) *zeteki*	Dyar	Culicini	0	0.0	0	0.0	0	0.0	0	0.0	0	0.0	22	0.7	6	0.3	2	0.1	3	0.1	0	0.0	0	0.0	0	0.0	0	0.0	25	0.8	5	0.1	3	0.1
*Culex* (*Phe*.) *airozai*	Lane	Culicini	0		0		0		0		2		0		4		1		0		3		0		0		2		0		4		4	0.1
*Lutzia bigoti*	(Bellardi)	Culicini	0	0.0	0	0.0	0	0.0	0	0.0	0	0.0	0	0.0	1	0.0	0	0.0	0	0.0	0	0.0	0	0.0	0	0.0	0	0.0	0	0.0	1	0.0	0	0.0
*Coquillettidia albicosta*	(Peryassú)	Mansoniini	3	0.1	1	0.0	4	0.2	0	0.0	1	0.1	415	12.4	13	0.6	13	0.7	36	1.4	11	0.7	1	0.0	1	0.0	11	0.3	5	0.2	11	0.3	476	8.7
*Coquillettidia arribalzagae*	(Theobald)	Mansoniini	0	0.0	0	0.0	0	0.0	0	0.0	1	0.1	5	0.2	12	0.5	0	0.0	15	0.6	1	0.1	0	0.0	1	0.0	0	0.0	1	0.0	5	0.1	27	0.5
*Coquillettidia chrysonotum*	(Peryassú)	Mansoniini	2	0.1	0	0.0	0	0.0	0	0.0	0	0.0	14	0.4	0	0.0	0	0.0	0	0.0	0	0.0	0	0.0	2	0.0	0	0.0	14	0.5	0	0.0	0	0.0
*Coquillettidia hermanoi*	(Lane and Coutinho)	Mansoniini	1	0.0	2	0.1	0	0.0	0	0.0	0	0.0	49	1.5	0	0.0	0	0.0	3	0.1	18	1.1	2	0.1	1	0.0	0	0.0	27	0.9	6	0.2	37	0.7
*Coquillettidia juxtamansonia*	(Chagas)	Mansoniini	0	0.0	11	0.4	3	0.1	0	0.0	1	0.1	40	1.2	155	6.7	20	1.0	14	0.5	1	0.1	8	0.3	6	0.1	1	0.0	187	6.3	5	0.1	38	0.7
*Coquillettidia lynchi*	(Shannon)	Mansoniini	0	0.0	0	0.0	0	0.0	0	0.0	0	0.0	0	0.0	15	0.6	0	0.0	9	0.3	0	0.0	0	0.0	0	0.0	0	0.0	2	0.1	8	0.2	14	0.3
*Coquillettidia nigricans*	(Coquillett)	Mansoniini	0	0.0	0	0.0	0	0.0	2	0.1	0	0.0	2	0.1	0	0.0	14	0.7	1	0.0	0	0.0	0	0.0	0	0.0	2	0.1	2	0.1	0	0.0	15	0.3
*Coquillettidia venezuelensis*	(Theobald)	Mansoniini	19	0.5	4	0.2	6	0.2	5	0.2	3	0.2	1085	32.3	78	3.4	39	2.0	856	32.4	4	0.2	3	0.1	18	0.3	19	0.4	332	11.2	29	0.8	1713	31.3
*Mansonia amazonensis*	(Theobald)	Mansoniini	2	0.1	0	0.0	2	0.1	0	0.0	4	0.2	0	0.0	0	0.0	0	0.0	0	0.0	1	0.1	4	0.2	1	0.0	3	0.1	0	0.0	1	0.0	0	0.0
*Mansonia flaveola*	(Coquillett)	Mansoniini	0	0.0	0	0.0	1	0.0	0	0.0	0	0.0	0	0.0	0	0.0	0	0.0	0	0.0	0	0.0	0	0.0	0	0.0	1	0.0	0	0.0	0	0.0	0	0.0
*Mansonia humeralis*	Dyar and Knab	Mansoniini	0	0.0	2	0.1	359	13.9	14	0.6	0	0.0	0	0.0	0	0.0	0	0.0	1	0.0	2	0.1	2	0.1	4	0.1	370	8.4	0	0.0	0	0.0	7	0.1
*Mansonia indubitans*	Dyar and Shannon	Mansoniini	19	0.5	22	0.9	93	3.6	139	5.8	156	8.6	5	0.2	9	0.4	8	0.4	133	5.0	62	3.7	18	0.7	201	3.2	209	4.7	5	0.2	24	0.7	187	3.4
*Mansonia pseudotitillans*	(Theobald)	Mansoniini	0	0.0	0	0.0	0	0.0	0	0.0	0	0.0	0	0.0	1	0.0	0	0.0	0	0.0	0	0.0	0	0.0	0	0.0	0	0.0	1	0.0	0	0.0	0	0.0
*Mansonia sp*.		Mansoniini	0	0.0	0	0.0	2	0.1	0	0.0	0	0.0	0	0.0	0	0.0	0	0.0	0	0.0	0	0.0	0	0.0	0	0.0	2	0.1	0	0.0	0	0.0	0	0.0
*Mansonia titillans*	(Walker)	Mansoniini	14	0.4	7	0.3	105	4.1	51	2.1	7	0.4	42	1.3	6	0.3	572	28.9	68	2.6	1	0.1	66	2.5	26	0.4	92	2.1	155	5.2	530	14.7	3	0.1
*Johnbelkinia longipes*	(Fabricius)	Sabethini	0	0.0	0	0.0	0	0.0	0	0.0	0	0.0	0	0.0	1	0.0	0	0.0	0	0.0	0	0.0	0	0.0	0	0.0	0	0.0	1	0.0	0	0.0	0	0.0
*Limatus durhami*	Theobald	Sabethini	0	0.0	0	0.0	0	0.0	0	0.0	0	0.0	0	0.0	1	0.0	0	0.0	0	0.0	3	0.2	0	0.0	0	0.0	0	0.0	0	0.0	1	0.0	3	0.1
*Trichoprosopon digitatum*	(Rondani)	Sabethini	0	0.0	0	0.0	0	0.0	0	0.0	0	0.0	0	0.0	0	0.0	1	0.1	2	0.1	2	0.1	0	0.0	0	0.0	0	0.0	0	0.0	0	0.0	5	0.1
*Trichoprosopon sp*.		Sabethini	0	0.0	0	0.0	0	0.0	0	0.0	0	0.0	1	0.0	1	0.0	0	0.0	1	0.0	3	0.2	0	0.0	0	0.0	0	0.0	1	0.0	1	0.0	4	0.1
*Wyeomyia sp*.		Sabethini	1	0.0	0	0.0	0	0.0	0	0.0	0	0.0	1	0.0	0	0.0	0	0.0	0	0.0	5	0.3	0	0.0	0	0.0	1	0.0	2	0.1	1	0.0	3	0.1
*Uranotaenia apicalis*	Theobald	Uranotaeniini	0	0.0	0	0.0	0	0.0	0	0.0	0	0.0	0	0.0	1	0.0	6	0.3	5	0.2	0	0.0	0	0.0	0	0.0	0	0.0	0	0.0	0	0.0	12	0.2
*Uranotaenia calosomata*	Dyar and Knab	Uranotaeniini	0	0.0	0	0.0	0	0.0	0	0.0	0	0.0	0	0.0	3	0.1	1	0.1	3	0.1	0	0.0	0	0.0	0	0.0	0	0.0	0	0.0	1	0.0	6	0.1
*Uranotaenia calosomata* (near)		Uranotaeniini	0	0.0	0	0.0	0	0.0	0	0.0	0	0.0	0	0.0	0	0.0	0	0.0	1	0.0	0	0.0	0	0.0	0	0.0	0	0.0	0	0.0	0	0.0	1	0.0
*Uranotaenia davisi*	Lane	Uranotaeniini	0	0.0	0	0.0	0	0.0	0	0.0	0	0.0	0	0.0	0	0.0	1	0.1	0	0.0	0	0.0	0	0.0	0	0.0	0	0.0	1	0.0	0	0.0	0	0.0
*Uranotaenia geometrica*	Theobald	Uranotaeniini	0	0.0	0	0.0	1	0.0	0	0.0	2	0.1	4	0.1	20	0.9	26	1.3	8	0.3	2	0.1	3	0.1	0	0.0	0	0.0	6	0.2	9	0.3	45	0.8
*Uranotaenia lowii*	Theobald	Uranotaeniini	0	0.0	0	0.0	0	0.0	0	0.0	1	0.1	0	0.0	5	0.2	0	0.0	0	0.0	0	0.0	0	0.0	0	0.0	1	0.0	0	0.0	0	0.0	5	0.1
*Uranotaenia nataliae*	Lynch Arribálzaga	Uranotaeniini	0	0.0	0	0.0	0	0.0	0	0.0	0	0.0	0	0.0	1	0.0	0	0.0	0	0.0	0	0.0	0	0.0	0	0.0	0	0.0	0	0.0	1	0.0	0	0.0
*Uranotaenia pulcherrima*	Lynch Arribálzaga	Uranotaeniini	0	0.0	0	0.0	0	0.0	0	0.0	0	0.0	0	0.0	2	0.1	5	0.3	6	0.2	1	0.1	0	0.0	0	0.0	0	0.0	0	0.0	1	0.0	13	0.2
*Uranotaenia sp*.		Uranotaeniini	0	0.0	0	0.0	0	0.0	0	0.0	0	0.0	0	0.0	0	0.0	0	0.0	2	0.1	0	0.0	0	0.0	0	0.0	0	0.0	0	0.0	0	0.0	2	0.0
**Total**			3996	100	2533	100	2592	100	2380	100	1823	100	3356	100	2330	100	1980	100	2642	100	1691	100	2612	100	6313	100	4410	100	2967	100	3603	100	5479	100

PLAND: forest cover percentage; HLC: human landing catch.

*Nyssorhynchus darlingi* was the dominant species in all landscape structure categories in the Campinarana vegetation class. The relative abundance of Anophelinae species was high in anthropogenic vegetation class in landscape A (> 70%), but lowest (< 20%) in landscapes B and D. Anophelinae abundance was higher than 50% in all landscape categories in vegetation classes defined as OTMF, OTMFA and TMF, with the exception of landscapes C and D for TMF (< 40%). The distribution of Culicidae groups by landscape structure and vegetation class is shown in the S5 Fig in S1 File.

### Landscape variables

The relationship between PLAND and ED (S6 Fig in S1 File) demonstrates that lower values of ED corresponded to either high or low PLAND percentages, because ED represents fragmentation levels in the landscape (r^2^ = 0.04; F_1,77_ = 3.2; *P* = 0.07). *Nyssorhynchus darlingi* was dominant in the peridomestic habitat (S7A Fig in S1 File); conversely Culicidae diversity was highest in the forest fringe (S7B Fig in S1 File) with the highest density of an individual species being *Coquillettidia venezuelensis*.

**Fig 3:**
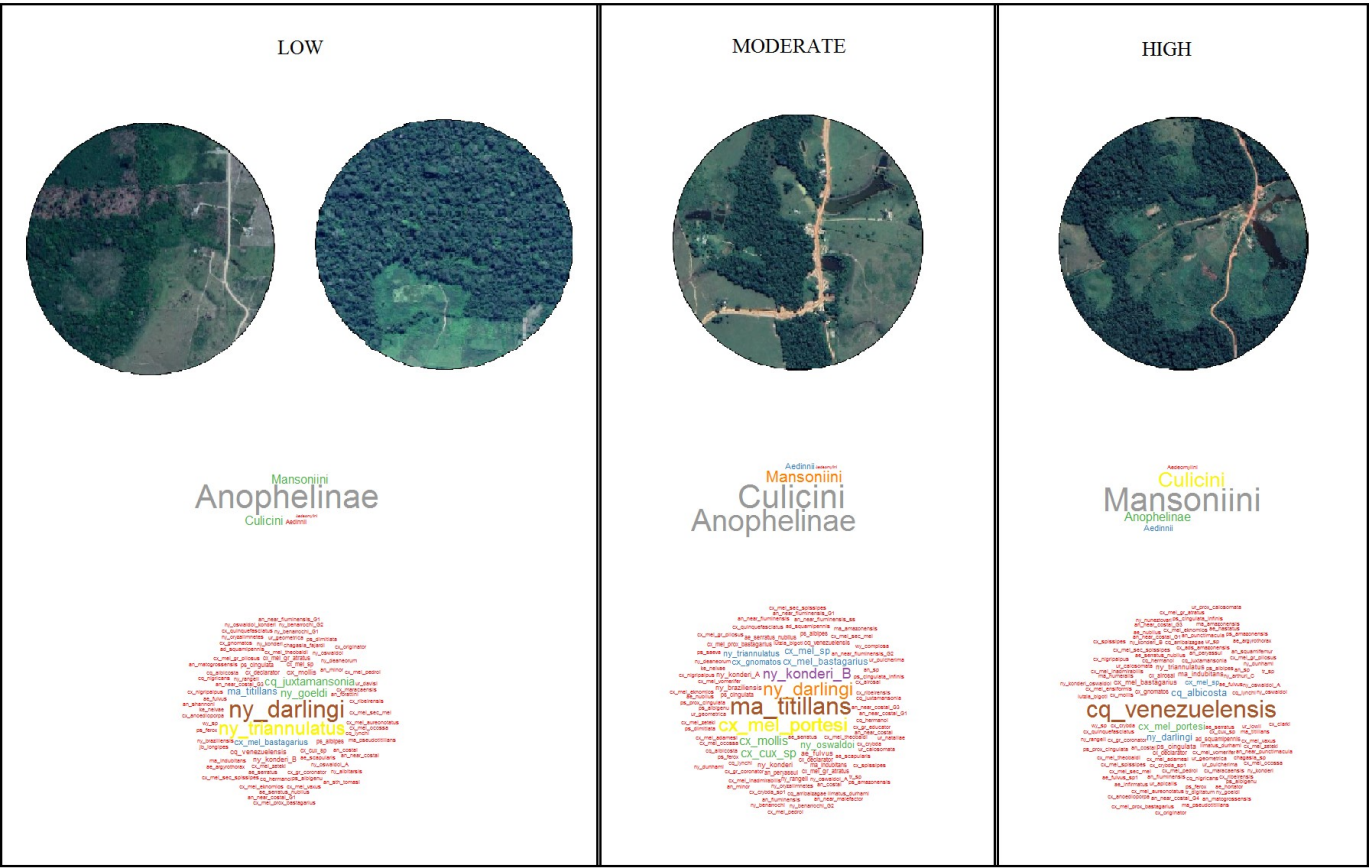
Culicidae species frequency displayed as a cloud for each of three ED categories (low, moderate and high) in forest fringe collections in rural areas of Amazonian Brazil. Font size is proportional to the frequency of Culicidae species. Edge density categories: Low (0–1.5 m / ha x 100); moderate (1.5–2.0 m / ha x 100); high (2.0–3.5 m / ha x 100).

The distribution of Culicidae species by ED categories (Fig 3) at the forest fringe demonstrates that *Ny*. *darlingi* was dominant in landscapes with a low edge density (0–1.5 m / ha x 100), *Mansonia titillans* in moderate (1.5–2.0 m / ha x 100), and *Cq*. *venezuelensis* in high edge density (2.0–3.5 m / ha x 100). Results of Tukey’s multiple comparison test showed significant differences in the Shannon-index (*P* = 0.04) between low (mean = 1.4) and moderate (mean = 1.7) edge densities (S8 Fig in S1 File). Differences in the Shannon-index were not statistically significant between other ED categories. *Nyssorhunchus darlingi* was dominant in all 79 peridomestic habitats sampled.

### Variables analysis

**Fig 4:**
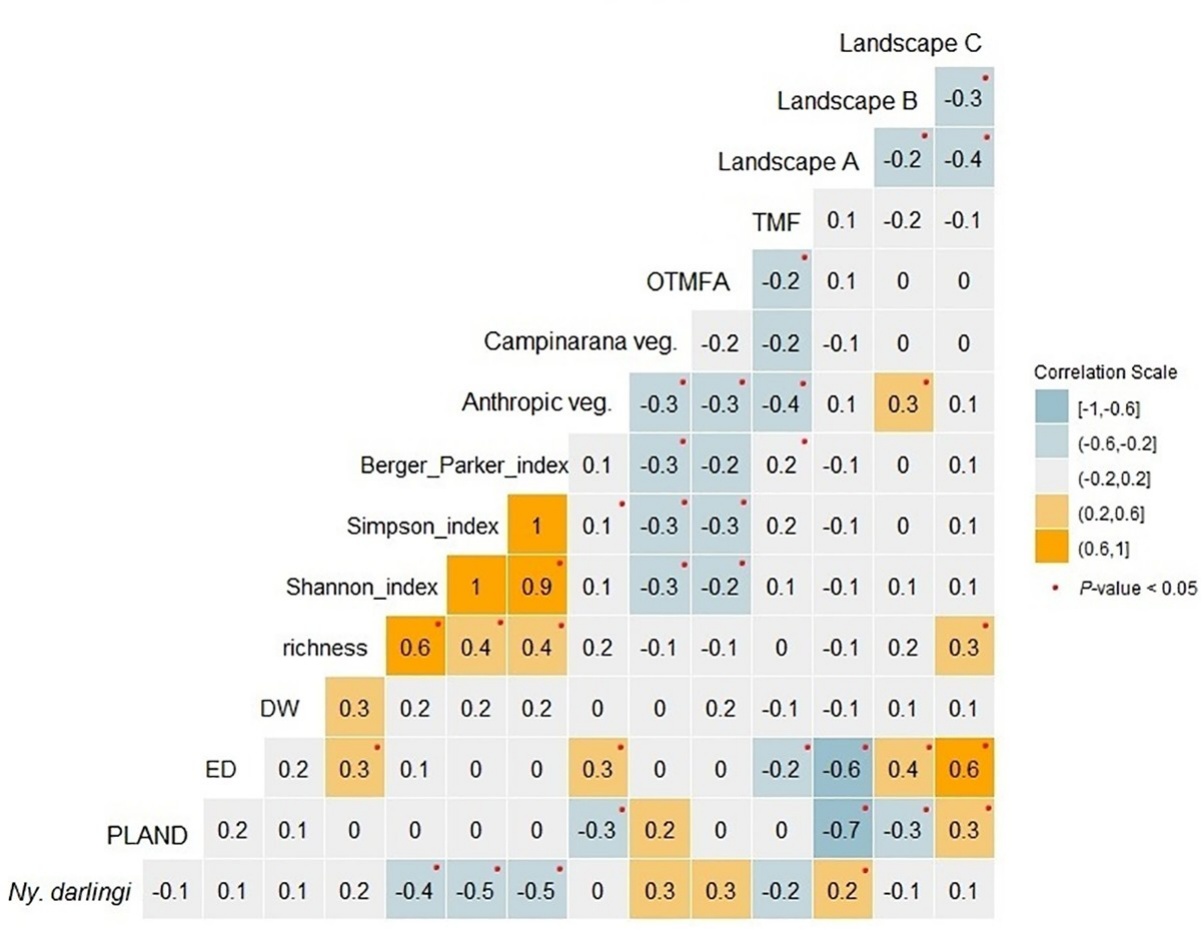
Correlation matrix among *Ny*. *darlingi* abundance, PLAND, ED, richness, DW, diversity indices (richness, Shannon, Simpson and Berger-Parker), Amazonian vegetation classes (Atp: Anthropogenic; OTMFA: Open Tropical Moist Forest with Arecaceae dominance; TMF: Tropical Moist Forest; Cpn: Campinarana), and landscape structures (A: PLAND ≤ 50% and ED < 0.015 m/ha; B: PLAND ≤ 50% and ED ≥ 0.015 m/ha; C: PLAND > 50% and ED ≥ 0.015 m/ha). PLAND: percentage of forest cover; ED: edge density; DW: distance of household from water drainage network.

Results of Spearman`s analysis showed that correlation among *Ny*. *darlingi* abundance and Shannon, Simpson and Berger-Parker indices were moderate and negative (Fig 4). Culicidae community richness showed a low, nonsignificant correlation with landscape components tested. Correlation between *Ny*. *darlingi* abundance and landscape A was low but significant (r = 0.2; *P* < 0.05).

The PCA results showed three dimensions of components for *Ny*. *darlingi* abundance variation, which explained 87.1% of the total variability observed (Dim.1 = 36.2%; Dim.2 = 28.9% and Dim.3 = 22.0%) (Table 2) in the data. The first component (Dim.1) showed a significant negative correlation between the Shannon-index and *Ny*. *darlingi* abundance (*P* < 0.01). The second (Dim.2) and third (Dim.3) dimensions represented the landscape variables attributed to PLAND and ED. The Dim.2 dimension showed a positive correlation between both PLAND and ED variables (*P* < 0.01) and *Ny*. *darlingi* abundance (r = 0.24; *P* = 0.03), and Dim.3, a positive correlation between ED and *Ny*. *darlingi* abundance (r = 0.29; *P* = 0.01), and negative for PLAND (S9 Fig in S1 File). The negative correlation in Dim.3 reinforced the relationship trends between PLAND, and ED variables showed in S6 Fig in S1 File.

**Table 2:** Principal components dimensions showed in PCA. A) open land (PLAND ≤ 50% and ED < 0.015 m/ha); B) fragmented open land (PLAND ≤ 50% and ED > 0.015 m/ha); C) fragmented forested land (PLAND ≤ 50% and ED > 0.015 m/ha); and D) forest cover (PLAND > 50% and ED < 0.015 m/ha).

	Components			Contributions		
Variables	Dim.1	Dim.2	Dim.3	Dim.1	Dim.2	Dim.3
Shannon-index	**0.84**			**48.2**		**45.0**
*Ny*. *darlingi* (abundance)	**- 0.80**			**44.7**		**39.0**
PLAND		**0.74**	**- 0.63**		**46.7**	
ED		**0.73**	**0.59**		**46.1**	
A	**- 0.79**	**- 1.41**				
B	**0.34**		**1.09**			
C		**0.93**				
D			**1.06**			
R^2^ (*P-*value)	0.14 (0.01)	0.64 (0.00)	0.68 (0.00)			
Cumulative percentage of variance	36.1	65.0	87.1			

In the forest fringe habitats, the difference in the Shannon index (mean = 1.7) calculated for fragmented forested landscape (C) and forested landscape (D) (mean = 1.2) was significant (*P* < 0.01). Differences between the Shannon index values calculated for fragmented open (B) and open (A) landscapes were not statistically significant (S10 Fig in S1 File).

Considering the overall mosquito data from the peridomestic (HLC) plus forest fringe (ST) habitats, the results of Kruskal-Wallis test showed that the differences in *Ny*. *darlingi* abundance between fragmented forested (C), forested (D), fragmented open (B) and open (A) landscapes were statistically significant (chi-squared = 9.9; *P* = 0.02). The Mann-Whitney-Wilcoxon test showed that difference in *Ny*. *darlingi* abundance found in the anthropogenic matrix (A) with low forest cover and low edge density, and the forested matrix (D) with high forest cover and low edge density, was statistically significant (*P* = 0.03). *Nyssorhynchus darlingi* showed higher abundance in anthropogenic matrix A (mean = 315 individuals) than in forest matrix D (mean = 62 individuals). Differences in the abundance of this species in fragmented open B (mean = 85 individuals) and fragmented forest landscape C (mean = 133 individuals) matrixes were nonsignificant (*P* > 0.05) (S11 Fig in S1 File).

As field collection localities were situated in areas with distinct vegetation classes (Fig 1), we tested their potential influence on Culicidae diversity and *Ny*. *darlingi* abundance using the Shannon-index. Results of a Tukey’s test showed that landscapes OTMFA had statistically lower Culicidae diversity than OTMF (*P* = 0.03). The sampling units in the Campinarana vegetation class showed a significant difference compared with those in TMF (*P* = 0.03) and OTMF (*P* = 0.01). Results of a Kruskal-Wallis test showed that differences in *Ny*. *darlingi* abundance between the sampling unit vegetation classes were statistically significant (chi-squared = 20.7; *P* < 0.001) (S12 Fig in S1 File). The Mann-Whitney-Wilcoxon test found statistically significant differences between *Ny*. *darlingi* abundance in Anthropogenic (Atp) and OTMF (*P* = 0.04), OTMF and Campinarana (Cpn) (*P* < 0.001), and between OTMF and OTMFA (*P* = 0.002). Campinarana and TMF showed the highest abundance averages, with 204 and 234 specimens collected, respectively.

Results of analysis employing GLMM (Table 3) revealed that increased diversity measured by the Shannon-index corresponded with a decrease of 226 specimens of *Ny*. *darlingi* (*P* < 0.001), and an increase in PLAND, with edge density as the random effect, led to a decrease of 533 (*P* = 0.003). In model 2, the effects of ED categories at the forest fringe (random effect) on *Ny*. *darlingi* abundance and the Shannon-index in (β_1_ = -218; *P* < 0.001) and PLAND (β_1_ = -337; *P* = 0.04) were significant. Results of analysis considering model 3 were similar to those obtained with model 2 (Table 3).

**Table 3:** Generalized linear mixed models results of *Ny*. *darlingi* abundance in function of the fixed effects of the Shannon-index, PLAND, ED, and house-water drainage network distance, with edge density (ED) categories as the random effect.

	SLR				Model 1 ^†^				Model 2 ^‡^				Model 3 ^§^			
	β_1_	Standard Error	t-value	*P*-value	β_1_	Standard Error	t-value	*P*-value	β_1_	Standard Error	t-value	*P*-value	β_1_	Standard Error	t-value	*P*-value
Shannon-index	-224.4	68.0	-3.2	0.001	-226.8	64.9	-3.4	< 0.001	-218.8	67.6	-3.2	0.001	-218.8	67.6	-3.2	0.001
PLAND*	-343.8	176.1	-1.9	0.05	-533.3	178.9	-2.9	0.003	-337.3	166.5	-2.0	0.04	-337.3	166.6	-2.0	0.04
ED**	-9321.7	7784.5	-1.2	0.2												
Distance house-water drainage	-0.1	0.2	-0.7	0.4	-0.1	0.2	-0.7	0.4	-0.08	0.2	-0.4	0.6	-0.08	0.2	-0.4	0.7

SLR: Simple Linear Regression; * PLAND: forest cover percentage

** ED: Edge density

^†^ Model 1: ED categories at 500 m from peridomestic as random effect

‡ Model 2: ED categories at 500 m from forest fringe habitat as random effect

^§^ Model 3: landscape structure categories (A, B, C, D) as random effect.

**Fig 5:**
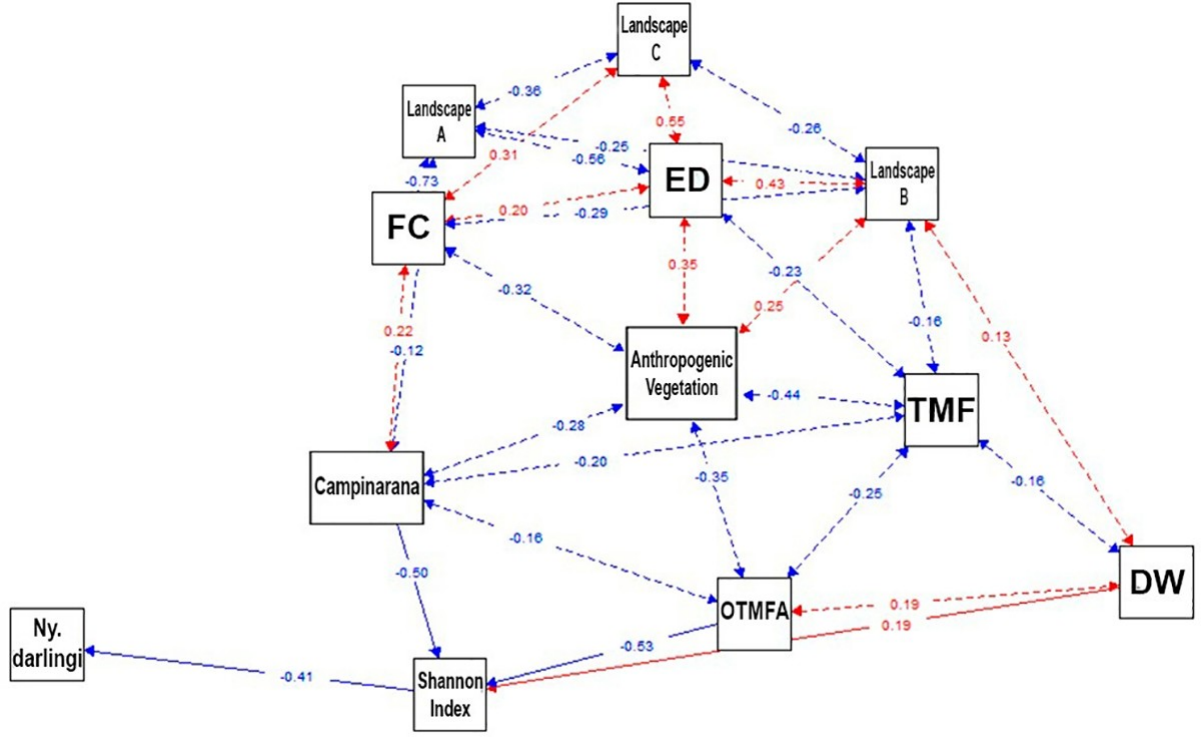
Structural equation model diagram to describes the relationships between changes in *Ny*. *darlingi* abundance with each independent variable: Shannon-index, PLAND, ED, landscape structure categories (A: PLAND ≤ 50% and ED < 0.015 m/ha; B: PLAND ≤ 50% and ED ≥ 0.015 m/ha; C: PLAND > 50% and ED ≥ 0.015 m/ha; D: PLAND > 50% and ED < 0.015 m/ha), Amazonian vegetation class (Atp: Anthropogenic; OTMF: Open Tropical Moist Forest; OTMFA: Open Tropical Moist Forest with Arecaceae dominance; TMF: Tropical Moist Forest; Cpn: Campinarana) and DW. Dashed lines represent a reciprocal path, and solid line one direction. Red arrows (positive coefficient) and blue arrows (negative coefficient) represent the significant path coefficients (*P* < 0.05).

The path model for *Ny*. *darlingi* explained 24% of the variation in abundance and 28% of the variation in the Shannon-index (Fig 5). There were strong negative effects of vegetation class OTMFA and Campinarana on the Shannon-index (-0.53 and -0.50, respectively). Anthropogenic vegetation showed moderate negative and significant reciprocal effects for OTMFA (r^2^ = -0.35), Campinarana (r^2^ = -0.28) and FC (r^2^ = -0.32). The results of path analysis are detailed in S3 Table in S1 File. The model showed a plausible causal direction on *Ny*. *darlingi* abundance that involves land use change, represented by negative effects of (1) Anthropogenic vegetation on FC, (2) Campinarana and OTMFA on the Shannon-index (mosquito community diversity). These effects can increase *Ny*. *darlingi* abundance. The forest loss process (deforestation), particularly in Campinarana and OTMFA, can decrease the diversity of the mosquito community and therefore increase *Ny*. *darlingi* abundance. The Path analysis results are presented in S4 Table in S1 File.

**Fig 6:**
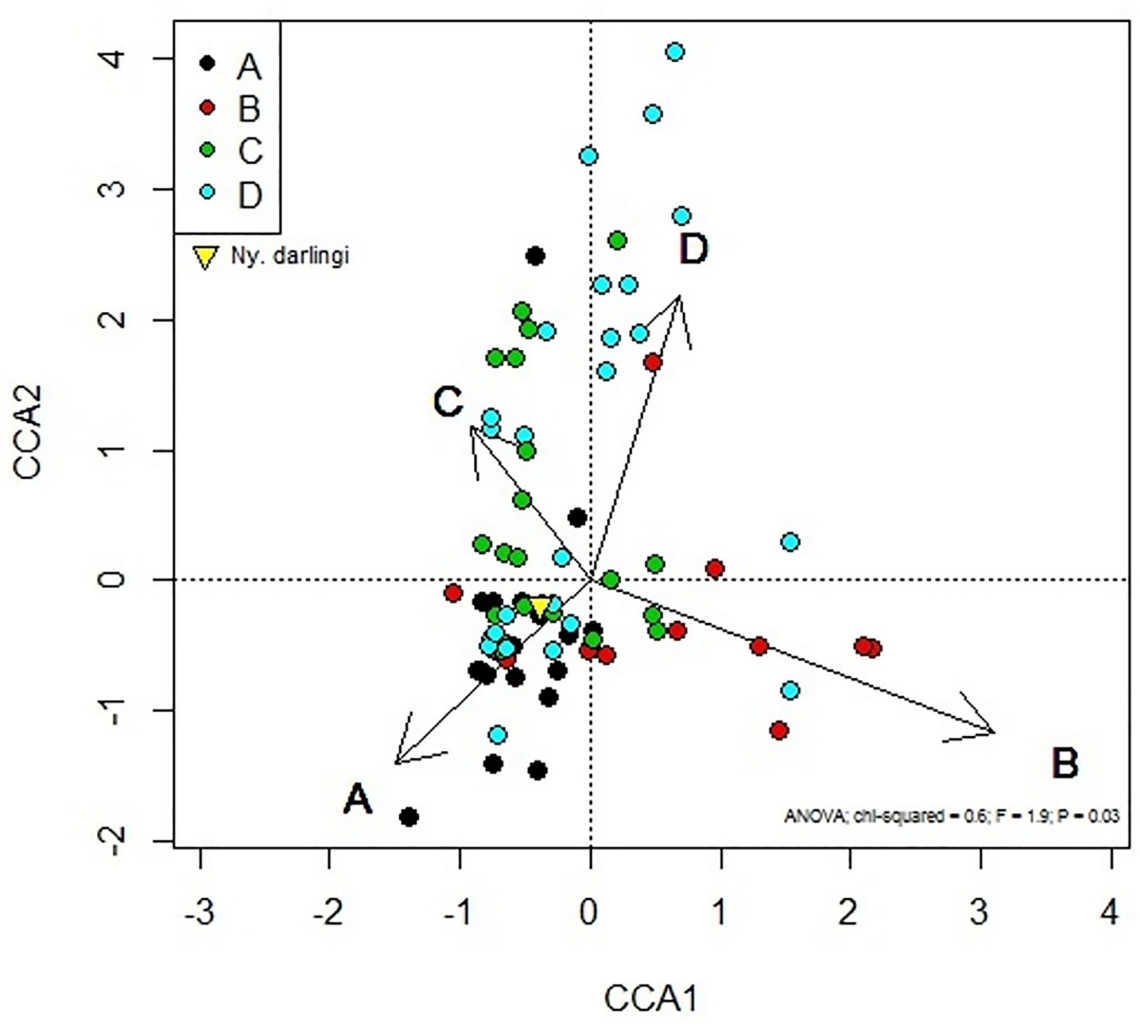
Canonical correspondence analysis of mosquito assemblages and landscape structure classifications at each sampling unit. Open landscape (A) is represented by black circles, fragmented open land (B) is represented by red, fragmented forest (C) is represented by green, and forested (D) is represented by blue circles. The length of the arrow representing the strength of the relationship. The yellow triangle represents *Ny*. *darlingi*. A: PLAND ≤ 50% and ED < 0.015 m/ha; B: PLAND ≤ 50% and ED ≥ 0.015 m/ha; C: PLAND > 50% and ED ≥ 0.015 m/ha; D: PLAND > 50% and ED < 0.015 m/ha.

The canonical correspondence analysis (Fig 6) demonstrated that the four landscape categories were pooled according to species composition of the 79 sampling units. In addition, *Ny*. *darlingi* abundance was associated with Culicidae communities represented by the landscapes A, C, and partially D, with the highest positive values associated with A. The components CCA1 and CCA2 explained 50% and 27% respectively, and together they explained 77% of the variation species-landscapes and 5.6% of the total data variation. Landscape B sampling units span the field collections, and landscape A was grouped on the CCA1 axis but separated out well on the CCA2 axis, suggesting that the lower forest cover habitat component is an important determinant of landscape A and B mosquito sample assemblages. The sampling units of landscape A were distinct, separating best both on the CCA1 and CCA2, suggesting that open habitat component was most important in determining landscape A mosquito assemblage.

## Discussion

Based on our results, it is possible to construct a network of anthropogenic changes and their effects on Culicidae diversity and *Ny*. *darlingi* in the natural tropical rainforest landscape. Here, the forest cover loss causes habitat loss and fragmentation, increasing the forest edge effects and changing the Culicidae species composition. Mosquito communities are strongly impacted by the mechanism associated with changes in land-use. Some species can readily invade new niches that emerged because of forest fragmentation and increased forest edges. High percentage of forest cover and increased Culicidae diversity can lead to a decrease of 533 and 226 mosquitoes, respectively. Deforestation leads to biodiversity loss [72], decreasing the dilution effect, and increasing diffuse competition. In these settings, the reduction of shading, raising the local temperature around human dwellings and in the neighboring larval habitats, reduces the larva to adult development time, and shifts primary vector species [3, 24, 35]. Therefore, habitat fragmentation is a key factor in the dynamics of this species because the increase in forest edge density facilitates the species dispersion and proliferation, creating a serious public health problem.

In Brazil, 99% of the 193,838 reported malaria cases in 2018 were in the Amazon biome, and more than 89% (173,006) were caused by *P*. *vivax* and 10% (19,283) by *P*. *falciparum* [73]. Mosquitoes of the subfamily Anophelinae are responsible for transmitting *Plasmodium* between infectious and susceptible humans. In endemic areas across the Amazon basin, malaria is associated with activities that require forest clearing, such as infrastructure construction, agricultural expansion, mining, logging, and increased urbanization [28, 74]. Increased malaria incidence is a trade-off of poorly planned or unplanned land occupation for socioeconomic development [32, 75–77].

It is well known that anthropogenic change in natural forest environments is a major driver of increased malaria incidence in subtropical and tropical endemic countries [30, 39, 78]. Recently, Chaves et al. (2018) demonstrated that increased malaria incidence is associated with complex mechanisms of change associated with deforestation, forest fragmentation, habitat degradation, geopolitical and social elements that facilitate malaria propagation. In addition, Chaves and collaborators [4] showed that an increase in the abundance of small patches of non-forest areas can intensify malaria transmission. Results of the current study provided strong statistical support for the mechanisms positively associated with anthropogenic modification in the Amazonian tropical rain forest landscape, changes in mosquito community, increased abundance of *Ny*. *darlingi* and intensification of malaria transmission. The socioecological determinants were not tested, however, despite being a corollary to mosquito bites, increasing vector abundance and putting settlers at risk of malaria [79], the socioeconomic conditions from localities were similar (i.e., subsistence rural practices in low populational density (< 1,500 people per km^2^), medium-low Human Development Index (HDI ~ 0.6), lack of basic sanitation and precarious housing). However, the landscape structure differed in each sampling unit of our study design, verifying that landscape (without socioeconomic confusion variables) operates as a regulator of the mosquito community and malaria risk.

Relative to the landscape habitat of the primary malaria vector, females will seek more shaded and safer larval habitats (realized niche) for egg laying [34]. In this context, moderate forest cover (i.e., a range between 45 ¬ 65%, partially shaded) increases the occurrence of larval habitats and adult resting places. On the other hand, low forest cover (full sunlight larval habitats) might pressure the species to disperse, because the females require appropriate water bodies (partially shaded) to deposit eggs. Some authors have demonstrated that the microclimate, pH, microbiota and other factors affect the productivity of *Ny*. *darlingi* larval habitats [41, 80, 81]. Results of the present study demonstrated that changes in the natural landscape structure, defined by the measurement of three metrics, significantly impacted adult productivity. Adult abundance is likely associated with an increase in the presence of suitable larval habitats and vertebrate hosts for blood feeding. Similarly, a positive relationship between clearing natural forest and the presence of *Ny*. *darlingi* was found in French Guiana [82].

Results of our study indicate that deforestation and forest clearing for human occupation and changes in land-use create new ecological and environmental conditions, which drive changes in the composition and community structure of plants [83, 84] and animals [85, 86], including insects of the family Culicidae. The anthropogenic changes in the tropical rainforest environment are linked to processes of ecological succession that can be initiated by a disturbance in a community [87]. Forest streams can be impacted by deforestation, i.e., once a tree is cut its trunk or branches can block the water flow, increasing resting places for adults, habitat for female oviposition, and microhabitat for immature development (S13 Fig in S1 File) [88].

Following deforestation, blocked water flow, and readily available blood meals, mosquitoes can proliferate. The introduction of new sources of blood for mosquitoes, and biotic components of malaria ecology such as ecological interactions among vector-host species also affect the dominance of vector species [56]. Humans and domestic animals, as well as local weather conditions are important factors in the changing landscape due to environmental disturbance [89, 90]. These factors together contribute to a dynamic process of change, dominated by fast-growing, resilient, generalist species that will be replaced by more competitive species as succession proceeds or new anthropogenic changes interfere in the natural succession process [91–93]. Medeiros-Sousa and colleagues [94] found strong evidence that biodiversity loss increases the risk of pathogen transmission.

This can be observed in mosquito communities, for example, when a taxonomic group increases or decreases its species assemblages in a chain of ecological succession. In our study, Culicidae diversity (β diversity) responded inversely to *Ny*. *darlingi* vector abundance across changes in PLAND metrics (S14 Fig in S1 File), a process that can influence diffuse competition and transmission dynamics of vector-borne pathogens [10]. This ecological process was used to explain malaria dynamics in the tropical rain forest, where access to vertebrate blood sources can be mediated by the threshold tolerance of a host to mosquito bites, and defensive host behavior [3]. The increased β diversity in higher PLAND can increase diffuse competition and decrease in malaria risk.

In S15 Fig in S1 File landscapes with less than 25% of PLAND had fewer rare species (richness) according to the collector curve effort. This evidence reinforces the hypothesis that forest cover can drive the dominance of *Ny*. *darlingi*. Therefore, if we define risk as the chance of a susceptible host being bitten by an infected vector, landscapes with lower forest cover percentages (< 25%) represent higher malaria risk in terms of human presence and a higher density of a competent vector species.

Overall, the results of the analyses showed that PLAND is a predictor variable for *Ny*. *darlingi* dominance in endemic areas of the Brazilian Amazon. Abundance of the mosquito vectors decreases with increase in the percent forest cover. Despite we have not directly tested the source-sink dynamics, the relationship between vector abundance and forest cover can also receive a contribution from this ecological range, as described by various authors [51, 52, 95]. In a malaria landscape where *Ny*. *darlingi* is the dominant vector, the source habitats are those that export individuals of the species, whereas the sink habitats import individuals, thereby sustaining the population in the heterogeneous landscape. The forest edge represents the source, whereas inside and nearby human dwellings represent the sink habitat for *Ny*. *darlingi*, with recolonization from sources due to a continuous process of increasing forest edge length by forest clearing and habitat fragmentation for human occupation and changes in land use. The establishment of a new *Ny*. *darlingi* population in a sink habitat is facilitated by the high reproductive success of the species, and rapid population growth in the Amazonian landscape. In addition, high temperature increases the development rate of immature stages as demonstrated by Chu et al. [35]. Populations in sink areas tend to occupy low-quality habitats that do not support them for long, whereas those in source areas, with high-quality habitat, persist longer and contribute to the recolonization of other fragments through dispersal of a fraction of the individuals [96].

Ecological factors that maintain *Ny*. *darlingi* in the peridomestic habitat (S7A Fig in S1 File) and the forest fringe (S7B Fig in S1 File) were impacted by PLAND and ED variables. The relationship between niche requirements and the patterns of landscape structure are supported by several studies of *Ny*. *darlingi* bionomics [21, 34, 97]. Therefore, considering the ecological determinants of the presence of *Ny*. *darlingi*, a low edge density can represents a source habitat because the availability of habitats for females to lay their eggs increases [34]. In contrast, a high edge density represents a sink habitat because the occurrence of habitat suitable for oviposition decreases [88]. In the peridomestic habitat, this mechanism is reversed, with *Ny*. *darlingi* the most abundant species. Likely, the anthropophilic/opportunistic blood-feeding behavior of the species facilitates the occurrence of part of the population in close contact with humans and domestic animals in an anthropogenic landscape. In Iquitos, Peruvian Amazon, the *Ny*. *darlingi* population is opportunistic, with a high proportion of the population feeding on humans (42.5%), and an unexpectedly 25.1% of individuals blood-feeding on avian hosts [98]. Similar results were found for *Ny*. *darlingi* collected in eastern Amazonian Brazil [99], where the availability of host species influences the blood feeding preference of the mosquito.

In conclusion, our study demonstrated that changes in Brazilian Amazon landscapes decrease overall Culicidae diversity and allow *Ny*. *darlingi* to become dominant if forest cover percentage decreases and edge density is low. Fragmented forest habitats inhabited by vulnerable people in precarious houses are the sources of this species, whereas continuous forested habitats or completely deforested habitats are sinks. The PLAND showed an empirical influence on blood-feeding activities and ED on oviposition behavior both lead to high *Ny*. *darlingi* abundance in the peridomestic habitat. Considering the key role of *Ny*. *darlingi* in malaria transmission cycle, our finding is highly relevant for understating heterogeneities in the dynamics of malaria epidemiology.

## Supporting information

S1 File (PDF)

## Abbreviations

PLAND: forest cover percentage
ED: edge density
HLC: human landing catch
ST: Shannon trap using light and human attraction
GLMM: generalized linear mixed model
H’: Shannon diversity index
DEH: dilution effect hypothesis
DCH: diffuse competition hypothesis
DW: water drainage network
DEM: Digital Elevation Model
PCA: Principal Component Analysis
SEM: Structural Equation Model
CCA: Canonical correspondence analysis
Atp: Anthropogenic
OTMF: Open Tropical Moist Forest
OTMFA: Open Tropical Moist Forest with Arecaceae dominance
TMF: Tropical Moist Forest
Cpn: Campinarana
