# Supplementary material for: Anthropogenic landscape decreases mosquito biodiversity and drives malaria vector proliferation in the Amazon rainforest
Source: PLoS One. 2021 Jan 14;16(1):e0245087. doi: 10.1371/journal.pone.0245087 (PMC7808592; doi:10.1371/journal.pone.0245087)
Supplement: S1 File — (PDF) [file pone.0245087.s001.pdf]

Anthropogenic landscape decreases mosquito biodiversity and drives malaria vector proliferation in the Amazon rainforest

Supporting information

Authors: Leonardo Suveges Moreira Chaves[*1]; Eduardo Sterlino Bergo[2]; Jan E. Conn[3,4]; Gabriel Zorello Laporta[5]; Paula Ribeiro Prist[6]; Maria Anice Mureb Sallum[&1]

[1] Departamento de Epidemiologia, Faculdade de Saúde Pública, Universidade de São Paulo, São Paulo, SP, Brazil.

[2] Superintendência de Controle de Endemias, Secretaria de Estado da Saúde de São Paulo, Araraquara-SP, Brazil.

[3] Wadsworth Center, New York State Department of Health, Albany, NY, USA.

[4] Department of Biomedical Sciences, School of Public Health, State University of New York, Albany, NY, USA.

[5] Setor de Pós-graduação, Pesquisa e Inovação, Centro Universitário Saúde ABC, Fundação ABC, Santo André, SP, Brazil.

[6] Department of Ecology, Institute of Bioscience, University of São Paulo, São Paulo, SP, Brazil.

[*] Corresponding author's e-mail: leonardosuveges@usp.br

[&] Senior author

Table of contents

SI 1: Field collection 3

SI 2: Study area and field collection 16

SI 3: Landscape metrics 17

SI 4: Descriptive analyses 18

SI 5: Link between Amazonian rural settlements and ecological succession. 33

SI 1: Field collection

Table SI 1. Social, ecological and climate characteristics for each field-collection locality.

**Sample**	**Col.**	**Munic.**	**State**	**Month-year**	**Season**	**PLAND**	**Landscape structure**[*]	**Veg. class** (IBGE, 2012)[†]	**Ecoregion** (Dinerstein *et al.*, 2017)	**Köppen & Geiger climate class**[‡]
1	1	Acrelândia	Acre	Jan-15	Wet	8%	A	Atp	Iquitos várzea	Am
2	1	Acrelândia	Acre	Jan-15	Wet	20%	A	TMF	Iquitos várzea	Am
3	1	Acrelândia	Acre	Jan-15	Wet	63%	C	OTMF	Iquitos várzea	Am
4	1	Acrelândia	Acre	Jan-15	Wet	70%	D	OTMF	Iquitos várzea	Am
5	1	Acrelândia	Acre	Jan-15	Wet	84%	D	OTMF	Iquitos várzea	Am
6	1	Acrelândia	Acre	Jan-15	Wet	93%	D	OTMF	Iquitos várzea	Am
7	2	Cruzeiro do Sul	Acre	Apr-15	wet-dry	46%	B	Atp	Southwest Amazon moist forests	Af
8	2	Cruzeiro do Sul	Acre	Apr-15	wet-dry	48%	B	Atp	Southwest Amazon moist forests	Af

Sample	Col.	Munic.	State	Month-year	Season	PLAND	Landscape structure[*]	Veg. class (IBGE, 2012)[†]	**Ecoregion** (Dinerstein *et al.*, 2017)	Köppen & Geiger climate class[‡]
9	2	Cruzeiro do Sul	Acre	Apr-15	wet-dry	51%	C	Atp	Southwest Amazon moist forests	Af
10	2	Cruzeiro do Sul	Acre	Apr-15	wet-dry	58%	C	Atp	Southwest Amazon moist forests	Af
11	2	Cruzeiro do Sul	Acre	Apr-15	wet-dry	81%	C	OTMFA	Southwest Amazon moist forests	Af
12	2	Cruzeiro do Sul	Acre	Apr-15	wet-dry	81%	C	Atp	Southwest Amazon moist forests	Af
13	3	Mâncio Lima	Acre	May-15	Dry	43%	B	Atp	Southwest Amazon moist forests	Af
14	3	Mâncio Lima	Acre	May-15	Dry	49%	B	Atp	Southwest Amazon moist forests	Af
15	3	Mâncio Lima	Acre	Jun-15	Dry	58%	C	Atp	Southwest Amazon	Af

**Sample**	**Col.**	**Munic.**	**State**	**Month-year**	**Season**	**PLAND**	**Landscape structure***	**Veg. class** (IBGE, 2012)†	**Ecoregion** (Dinerstein *et al.*, 2017)	**Köppen & Geiger climate class**‡
									moist forests	
16	3	Mâncio Lima	Acre	May-15	Dry	68%	C	TMF	Southwest Amazon moist forests	Af
17	3	Mâncio Lima	Acre	Jun-15	Dry	72%	C	OTMFA	Southwest Amazon moist forests	Af
18	3	Mâncio Lima	Acre	Jun-15	Dry	79%	C	Cpn	Southwest Amazon moist forests	Af
19	4	Lábrea	Amazonas	Aug-15	Dry	19%	A	Atp	Purus-Madeira moist forests	Am
20	4	Lábrea	Amazonas	Aug-15	Dry	31%	A	Atp	Purus-Madeira moist forests	Am
21	4	Lábrea	Amazonas	Aug-15	Dry	59%	C	OTMFA	Purus-Madeira moist forests	Am

Sample	Col.	Munic.	State	Month-year	Season	PLAND	Landscape structure*	Veg. class (IBGE, 2012)†	Ecoregion (Dinerstein *et al.*, 2017)	Köppen & Geiger climate class‡
22	4	Lábrea	Amazonas	Jul-15	Dry	67%	C	Atp	Purus-Madeira moist forests	Am
23	4	Lábrea	Amazonas	Aug-15	Dry	77%	D	OTMFA	Purus-Madeira moist forests	Am
24	4	Lábrea	Amazonas	Aug-15	Dry	87%	D	OTMFA	Purus-Madeira moist forests	Am
25	5	Acrelândia	Acre	Aug-15	Dry	9%	A	Atp	Purus-Madeira moist forests	Am
26	5	Acrelândia	Acre	Aug-15	Dry	9%	A	TMF	Purus-Madeira moist forests	Am
27	5	Acrelândia	Acre	Jan-15	Dry	64%	C	OTMF	Purus-Madeira moist forests	Am
28	5	Acrelândia	Acre	Aug-15	Dry	84%	D	OTMF	Purus-Madeira	Am

Sample	Col.	Munic.	State	Month-year	Season	PLAND	Landscape structure*	Veg. class (IBGE, 2012)†	Ecoregion (Dinerstein *et al*., 2017)	Köppen & Geiger climate class‡
									moist forests	
29	5	Acrelândia	Acre	Aug-15	Dry	84%	D	OTMF	Purus-Madeira moist forests	Am
30	5	Acrelândia	Acre	Aug-15	Dry	93%	D	OTMF	Purus-Madeira moist forests	Am
31	6	Machadinho D'Oeste	Rondônia	Oct-15	dry-wet	27%	B	OTMFA	Madeira-Tapajós moist forests	Am
32	6	Machadinho D'Oeste	Rondônia	Oct-15	dry-wet	34%	A	OTMFA	Madeira-Tapajós moist forests	Am
33	6	Machadinho D'Oeste	Rondônia	Oct-15	dry-wet	35%	B	OTMFA	Madeira-Tapajós moist forests	Am
34	6	Machadinho D'Oeste	Rondônia	Oct-15	dry-wet	38%	A	OTMFA	Madeira-Tapajós moist forests	Am

**Sample**	**Col.**	**Munic.**	**State**	**Month-year**	**Season**	**PLAND**	**Landscape structure***	**Veg. class** (IBGE, 2012)†	**Ecoregion** (Dinerstein *et al.*, 2017)	**Köppen & Geiger climate class**‡
35	6	Machadinho D'Oeste	Rondônia	Oct-15	dry-wet	46%	A	OTMFA	Madeira-Tapajós moist forests	Am
36	6	Machadinho D'Oeste	Rondônia	Oct-15	dry-wet	58%	D	OTMFA	Madeira-Tapajós moist forests	Am
37	7	Pacajá	Pará	Apr-16	wet-dry	25%	A	Atp	Xingu-Tocantins-Araguaia moist forests	Am
38	7	Pacajá	Pará	Apr-16	wet-dry	49%	B	TMF	Xingu-Tocantins-Araguaia moist forests	Am
39	7	Pacajá	Pará	Apr-16	wet-dry	53%	D	TMF	Xingu-Tocantins-Araguaia moist forests	Am
40	7	Pacajá	Pará	Apr-16	wet-dry	66%	D	TMF	Xingu-Tocantins-Araguaia	Am

Sample	Col.	Munic.	State	Month-year	Season	PLAND	Landscape structure*	Veg. class (IBGE, 2012)†	Ecoregion (Dinerstein *et al.*, 2017)	Köppen & Geiger climate class‡
									moist forests	
41	7	Pacajá	Pará	Apr-16	wet-dry	69%	D	TMF	Xingu-Tocantins-Araguaia moist forests	Am
42	7	Pacajá	Pará	Apr-16	wet-dry	79%	D	TMF	Xingu-Tocantins-Araguaia moist forests	Am
43	8	Humaitá	Amazonas	Jul-16	Dry	11%	A	Atp	Purus-Madeira moist forests	Am
44	8	Humaitá	Amazonas	Jul-16	Dry	21%	A	Atp	Purus-Madeira moist forests	Am
45	8	Humaitá	Amazonas	Jul-16	Dry	30%	A	OTMFA	Purus-Madeira moist forests	Am
46	8	Humaitá	Amazonas	Jul-16	Dry	48%	A	Atp	Purus-Madeira	Am

Sample	Col.	Munic.	State	Month-year	Season	PLAND	Landscape structure*	Veg. class (IBGE, 2012)†	Ecoregion (Dinerstein *et al.*, 2017)	Köppen & Geiger climate class‡
									moist forests	
47	8	Humaitá	Amazonas	Jul-16	Dry	57%	D	Atp	Purus-Madeira moist forests	Am
48	8	Humaitá	Amazonas	Jul-16	Dry	58%	C	OTMFA	Purus-Madeira moist forests	Am
49	9	Itacoatiara	Amazonas	Nov-16	Wet	39%	B	Atp	Uatumã-Trombetas moist forests	Af
50	9	Itacoatiara	Amazonas	Nov-16	Wet	53%	C	TMF	Uatumã-Trombetas moist forests	Af
51	9	Itacoatiara	Amazonas	Nov-16	Wet	66%	D	Atp	Uatumã-Trombetas moist forests	Af
52	9	Itacoatiara	Amazonas	Nov-16	Wet	73%	D	TMF	Uatumã-Trombetas moist forests	Af

Sample	Col.	Munic.	State	Month-year	Season	PLAND	Landscape structure*	Veg. class (IBGE, 2012)†	Ecoregion (Dinerstein *et al.*, 2017)	Köppen & Geiger climate class‡
53	9	Itacoatiara	Amazonas	Nov-16	Wet	77%	D	TMF	Uatumã-Trombetas moist forests	Af
54	9	Itacoatiara	Amazonas	Nov-16	Wet	78%	D	TMF	Uatumã-Trombetas moist forests	Af
55	10	Rodrigues Alves	Acre	Jun-17	Dry	24%	B	Atp	Iquitos várzea	Af
56	10	Rodrigues Alves	Acre	Jul-17	Dry	29%	B	Atp	Iquitos várzea	Af
57	10	Rodrigues Alves	Acre	Jun-17	Dry	64%	C	TMF	Iquitos várzea	Af
58	10	Rodrigues Alves	Acre	Jun-17	Dry	70%	C	TMF	Iquitos várzea	Af
59	11	Cruzeiro do Sul	Acre	Jul-17	Dry	31%	B	Atp	Southwest Amazon moist forests	Af
60	11	Guajará	Amazonas	Jul-17	Dry	52%	C	Atp	Southwest Amazon moist forests	Af
61	11	Guajará	Amazonas	Jul-17	Dry	64%	C	Atp	Southwest Amazon	Af

**Sample**	**Col.**	**Munic.**	**State**	**Month-year**	**Season**	**PLAND**	**Landscape structure**[*]	**Veg. class** (IBGE, 2012)[†]	**Ecoregion** (Dinerstein *et al.*, 2017)	**Köppen & Geiger climate class**[‡]
									moist forests	
62	11	Cruzeiro do Sul	Acre	Jul-17	Dry	67%	C	Atp	Southwest Amazon moist forests	Af
63	11	Guajará	Amazonas	Jul-17	Dry	70%	C	Atp	Southwest Amazon moist forests	Af
64	11	Cruzeiro do Sul	Acre	Jul-17	Dry	74%	D	Atp	Southwest Amazon moist forests	Af
65	11	Cruzeiro do Sul	Acre	Jul-17	Dry	77%	C	Atp	Southwest Amazon moist forests	Af
66	11	Guajará	Amazonas	Jul-17	Dry	83%	D	TMF	Southwest Amazon moist forests	Af
67	12	Presidente Figueiredo	Amazonas	Aug-17	Dry	13%	A	TMF	Monte Alegre várzea	Af

Sample	Col.	Munic.	State	Month-year	Season	PLAND	Landscape structure*	Veg. class (IBGE, 2012)†	Ecoregion (Dinerstein *et al.*, 2017)	Köppen & Geiger climate class‡
68	12	Presidente Figueiredo	Amazonas	Aug-17	Dry	18%	A	TMF	Monte Alegre várzea	Af
69	12	Presidente Figueiredo	Amazonas	Aug-17	Dry	24%	A	TMF	Monte Alegre várzea	Af
70	12	Presidente Figueiredo	Amazonas	Aug-17	Dry	29%	A	Atp	Monte Alegre várzea	Af
71	12	Presidente Figueiredo	Amazonas	Aug-17	Dry	30%	A	TMF	Monte Alegre várzea	Af
72	12	Presidente Figueiredo	Amazonas	Aug-17	Dry	72%	D	Cpn	Monte Alegre várzea	Af
73	13	São Gabriel da Cachoeira	Amazonas	Nov-17	Wet	40%	B	Cpn	Negro-Branco moist forests	Af
74	13	São Gabriel da Cachoeira	Amazonas	Nov-17	Wet	46%	A	Cpn	Negro-Branco moist forests	Af
75	13	São Gabriel da Cachoeira	Amazonas	Nov-17	Wet	69%	D	Cpn	Negro-Branco	Af

**Sample**	**Col.**	**Munic.**	**State**	**Month-year**	**Season**	**PLAND**	**Landscape structure***	**Veg. class** (IBGE, 2012)†	**Ecoregion** (Dinerstein *et al.*, 2017)	**Köppen & Geiger climate class‡**
									moist forests	
76	13	São Gabriel da Cachoeira	Amazonas	Nov-17	Wet	73%	D	Cpn	Negro-Branco moist forests	Af
77	13	São Gabriel da Cachoeira	Amazonas	Nov-17	Wet	76%	C	Cpn	Negro-Branco moist forests	Af
78	13	São Gabriel da Cachoeira	Amazonas	Nov-17	Wet	78%	D	Cpn	Negro-Branco moist forests	Af
79	13	São Gabriel da Cachoeira	Amazonas	Nov-17	Wet	83%	D	Cpn	Negro-Branco moist forests	Af

* A: open land (PLAND ≤ 50 % and ED < 0.015 m/ha), B: fragmented open land (PLAND ≤ 50 % and ED ≥ 0.015 m/ha), C: fragmented forested land (PLAND > 50 % and ED ≥ 0.015 m/ha), and D: forested land (PLAND > 50 % and ED < 0.015 m/ha);

† Atp: Anthropogenically-modified vegetation, OTMF: Open Tropical Moist Forest, OTMFA: Open Tropical Moist Forest with Arecaceae dominance, TMF: Tropical Moist Forest, and Cpn: Campinarana;

‡ Af: Tropical Rainforest, Am: Tropical Monsoon, As: Tropical wet and dry, Aw: Savanna, Cfa: Humid subtropical, and Cwa: Subtropical-dry winter.Col. – Collection Number; Munc. – Municipality; Veg. – Vegetation; IBGE – Brazilian Institute of Geography and Statistics (1).

SI 2: Study area and field collection

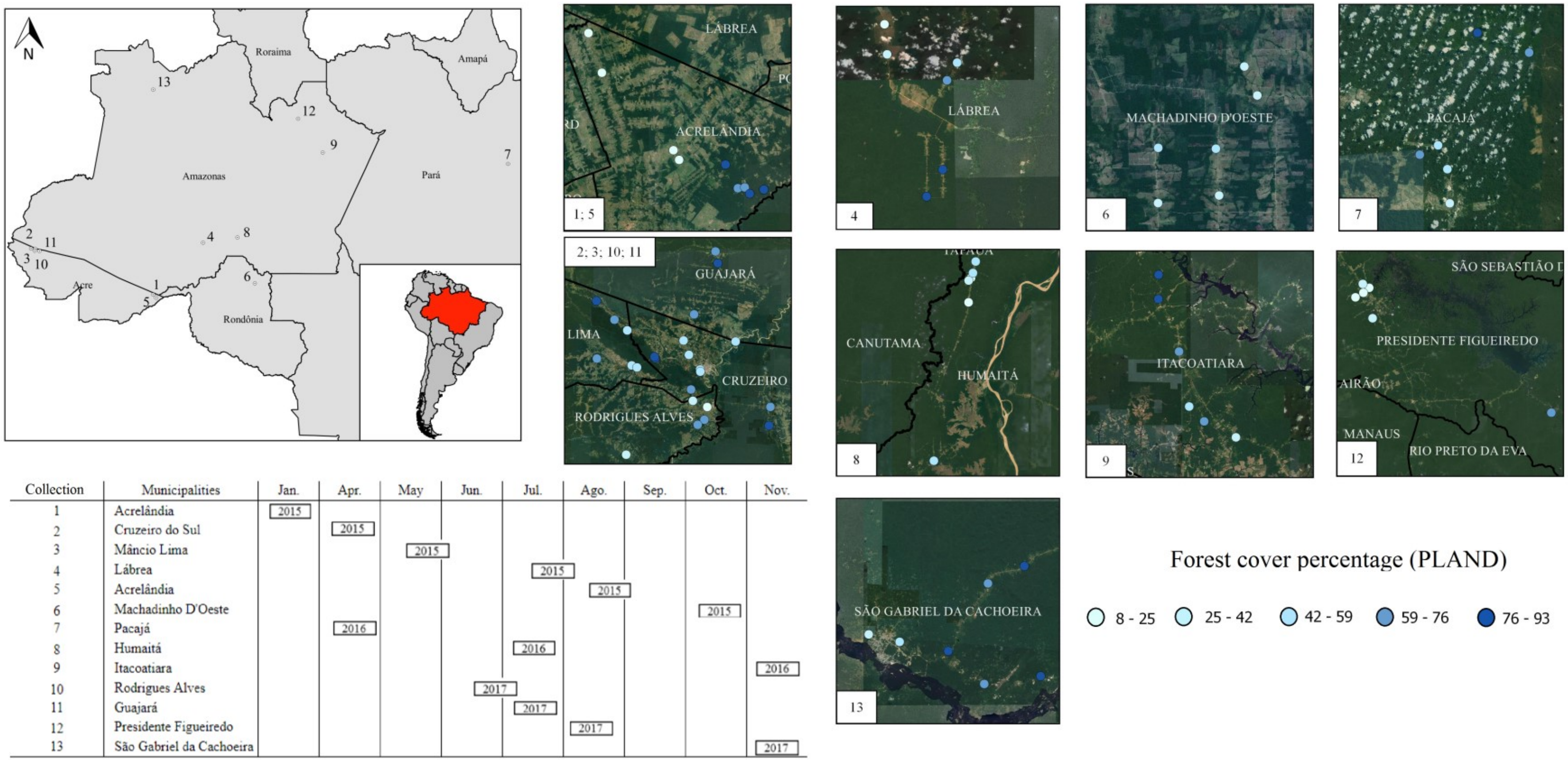

Collection	Municipalities	Jan.	Apr.	May	Jun.	Jul.	Ago.	Sep.	Oct.	Nov.
1	Acrelândia	2015								
2	Cruzeiro do Sul		2015							
3	Mâncio Lima			2015						
4	Lábrea					2015				
5	Acrelândia						2015			
6	Machadinho D'Oeste								2015	
7	Pacajá		2016							
8	Humaitá					2016				
9	Itacoatiara									2016
10	Rodrigues Alves				2017					
11	Guajará					2017				
12	Presidente Figueiredo						2017			
13	São Gabriel da Cachoeira									2017

Figure SI 1. Study area and detailed field collection localities including forest cover level in the Brazilian Amazon. The table includes collection number, municipality, and collection month and year. Numbers in each square refer to map location and collection number. The ecological features for each field collection locality are presented in supplementary Table SI 1. Satellite images obtained from Google Earth Pro© under a CC BY license, with permission from PLOS ONE, original copyright 2020. Reprinted from QGIS version 2.8 without any changes, under a CC BY license, with permission from PLOS ONE, original copyright 2020.

SI 3: Landscape metrics

PLAND was calculated with the formula: PLAND = $P_i = \frac{\sum_{j=1}^{n} a_{ij}}{A}$ (100), where

Pi = the proportion of forest per sampling unit;

aij = the area of each forest cover;

i = land-use class (2 classes: forest and non-forest pixels);

j = number of pixels; and

A = the total area of the landscape under analysis.

Each landscape can have one or more fragments and the edge density (ED) per fragment was calculated with the following formula (2):

ED = $\frac{\sum_{k=1}^{m'} e_{ik}}{A}$ (10,000);

m' = number of patch types (classes) present in the landscape, including the landscape border if present;

e_{ik} = the total length (m) of the edge that surrounds each forest cover patch of the landscape;

A = the total area of the landscape (m^2).

SI 4: Descriptive analyses

Table SI 2. List of all models analyzed in the regression analysis for each spatial scale arrangement. SLR – Simple linear regression; PV – Predictor variable; RV – Random variable.

**Model Name**	**Response Variable**	**Predictor Variables**	**Random variable**	**Scale (m)**
SLR 1	*Ny. darlingi* abundance	Shannon-index	None	1,000
SLR 2	*Ny. darlingi* abundance	PLAND	None	1,000
SLR 3	*Ny. darlingi* abundance	ED	None	1,000
SLR 4	*Ny. darlingi* abundance	DW	None	1,000
Model 1	*Ny. darlingi* abundance	Shannon-index + PLAND + DW	ED (peridomestic habitat)	1,000 (PV); 500 (RV)
Model 2	*Ny. darlingi* abundance	Shannon-index + PLAND + DW	ED (forest fringe habitat)	1,000 (PV); 500 (RV)
Model 3	*Ny. darlingi* abundance	Shannon-index + PLAND + DW	Landscape categories A, B, C, D	1,000

Table SI 3. Sample distribution according to landscape structure and vegetation class variables.

**Landscape structure**	*Ny. darlingi* abundance	**PLAND (%)**	**ED m/ha**	**DW (m)**	**Samples**	**Atp**	**OTMF**	**OTMFA**	**TMF**	**Cpn**	**Sampling unit distribution by vegetation profile**<br>**Atp**	**OTMF**	**OTMFA**	**TMF**	**Cpn**
Landscape A (PLAND ≤ 50 % and ED < 0.015 m/ha)	315.0 (-4.7 – 634.7)	0.25 (0.19 – 0.31)	0.010 (0.008 – 0.011)	189.61 (86.4 – 292.8)	20	6	0	3	9	2					
Landscape B (PLAND ≤ 50 % and ED ≥ 0.015 m/ha)	85.4 (19.7 - 151.0)	0.38 (0.32 - 0.44)	0.020 (0.017 – 0.023)	251.90 (102.6 – 401.1)	12	4	3	2	2	1					
Landscape C (PLAND > 50 % and ED ≥ 0.015 m/ha)	133.6 (67.9 - 199.2)	0.66 (0.61 - 0.70)	0.019 (0.018 – 0.021)	193.75 (131.6 – 255.8)	22	3	5	5	4	5					
Landscape D (PLAND > 50 % and ED < 0.015 m/ha)	61.8 (21.6 - 102.0)	0.75 (0.70 - 0.79)	0.012 (0.011 – 0.013)	176.15 (107.3 – 245.0)	25	17	0	3	4	1					
Total					79	30	8	13	19	9					

**Vegetation profile**	*Ny. darlingi* abundance	**PLAND (%)**	**ED m/ha**	**DW (m)**	**Samples**	**A**	**B**	**C**	**D**	**Sampling unit distribution by landscape structure**<br>**A**	**B**	**C**	**D**
Atp (Anthrogenic)	111.0 (55.1 – 166.9)	0.45 (0.37 – 0.53)	0.017 (0.015 – 0.019)	192.77 (127.91 – 257.63)	30	9	8	10	3				
OTMF (Open Tropical Moist Forest)	12.7 (-0.18 – 25.7)	0.80 (0.70 – 0.90)	0.014 (0.013 – 0.015)	190.0 (53.08 – 326.74)	8	0	0	2	6				
OTMFA (Open Tropical Moist Forest - Arecaceae dominance)	160.3 (79.0 – 241.7)	0.54 (0.41 – 0.66)	0.014 (0.012 – 0.016)	273.86 (118.66 – 429.05)	13	4	2	4	3				
TMF (Tropical Moist Forest)	234.2 (-107.4 – 575.9)	0.52 (0.40 – 0.64)	0.013 (0.010 – 0.016)	144.09 (84.56 – 203.61)	19	6	1	4	8				
Cpn (Campinarana)	204.5 (85.2 – 323.9)	0.68 (0.57 – 0.80)	0.014 (0.011 – 0.017)	209.01 (37.46 – 380.55)	9	1	1	2	5				
Total					79	20	12	22	25				

NB. See Table SI 1 for definitions of vegetation profile.

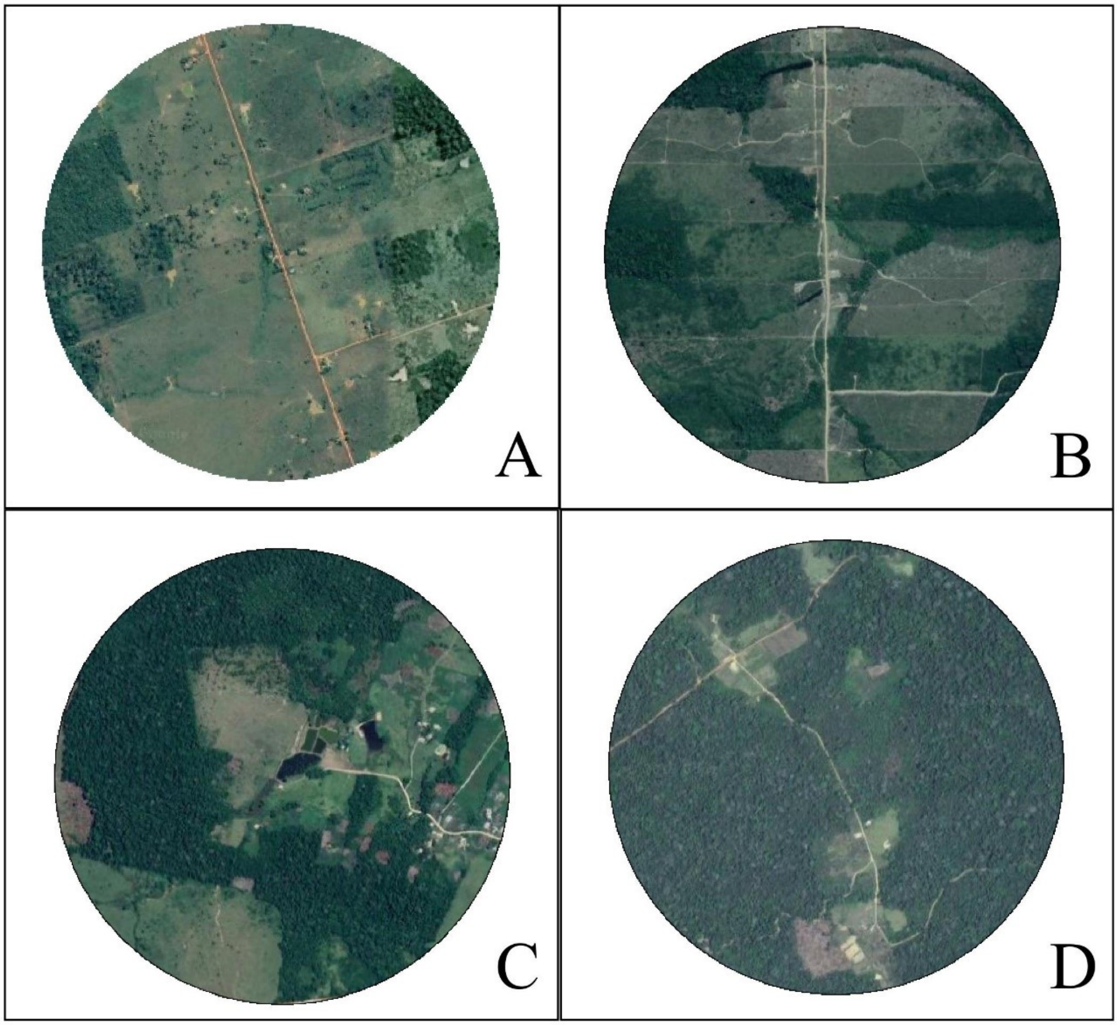

Figure SI 2. Landscape structure categories. Localities: A) Acrelândia/AC (collection 5); B) Machadinho D'Oeste/RO (collection 6); C) Mâncio Lima/AC (collection 3); D) Acrelândia/AC (collection 1). Reprinted from QGIS version 2.8 without any changes, under a CC BY license, with permission from PLOS ONE, original copyright 2020. Satellite images reprinted from Google Earth Pro© under a CC BY license, with permission from PLOS ONE, original copyright 2020.

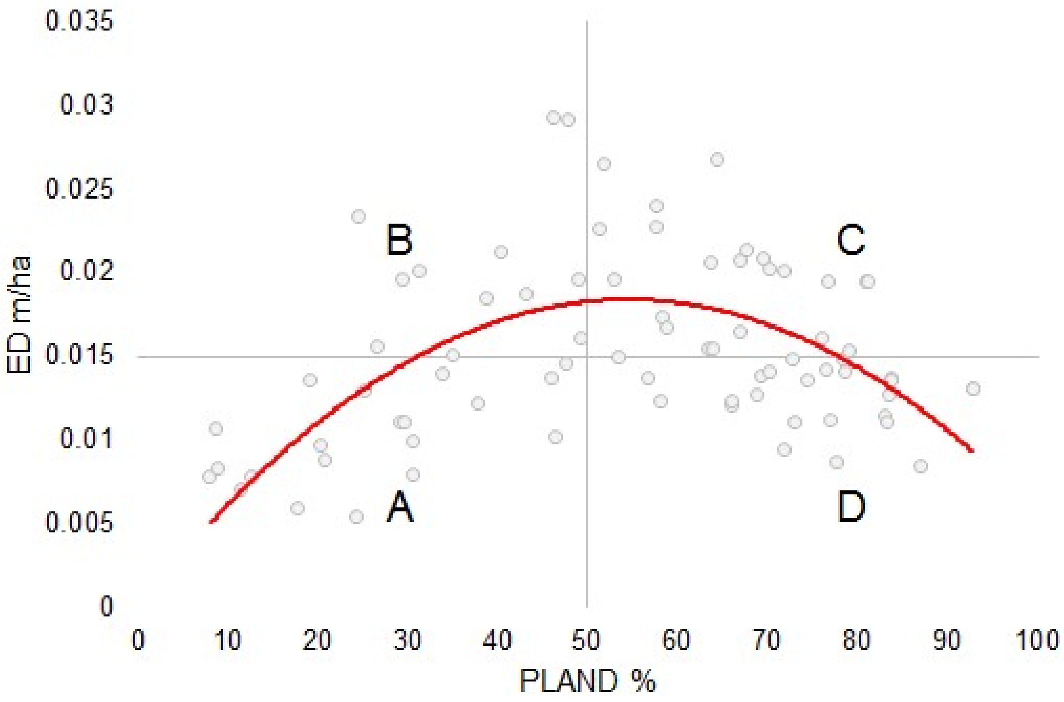

Figure SI 3. Distribution of collection points (grey circles) according to the relationship of PLAND and ED (red line), i.e., landscape structure categories. A: PLAND ≤ 50 % and ED < 0.015 m/ha; B: PLAND ≤ 50 % and ED ≥ 0.015 m/ha; C: PLAND > 50 % and ED ≥ 0.015 m/ha; D: PLAND > 50 % and ED < 0.015 m/ha.

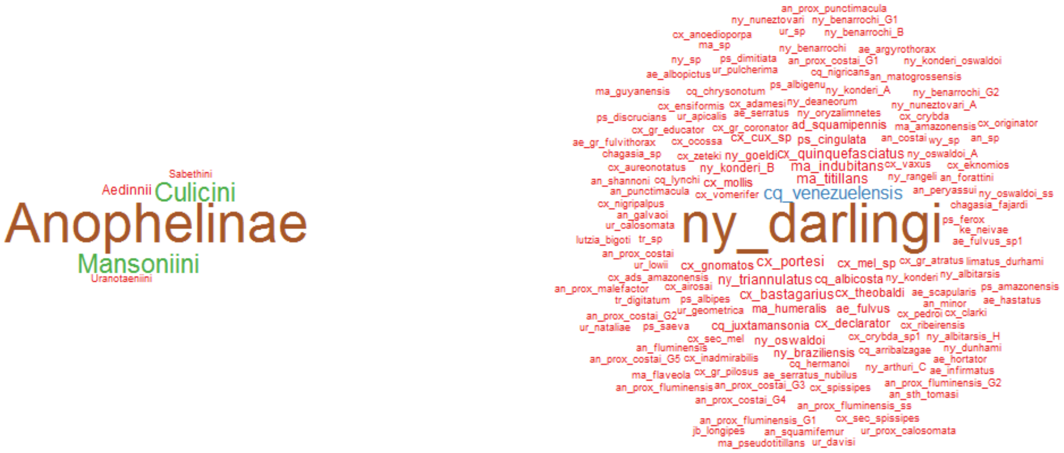

Figure SI 4. Culicidae taxonomic units (left cloud) and species frequency (right cloud) displayed as a cloud for a total of 79-sampling units in Amazonian Brazil. Font size is proportional to the frequency of Culicidae species.

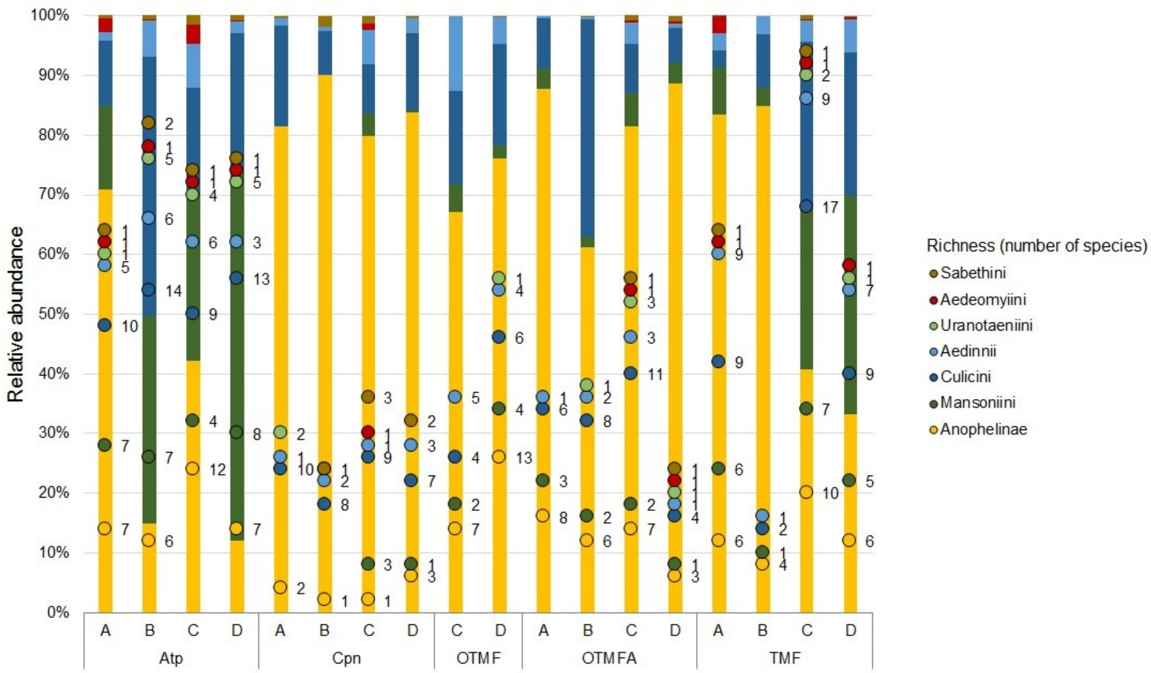

Figure SI 5. Relative abundance percentage (bars) and richness (circles) of Culicidae collected using HLC and ST, in landscapes structures (A, B, C and D) in five Amazonian vegetation class (Atp, Cpn, OTMF, OTMFA, TMF). Landscapes - A: PLAND ≤ 50 % and ED < 0.015 m/ha; B: PLAND ≤ 50 % and ED ≥ 0.015 m/ha; C: PLAND > 50 % and ED ≥ 0.015 m/ha; D: PLAND > 50 % and ED < 0.015 m/ha. Vegetation class - Atp: Anthropogenic; OTMF: Open Tropical Moist Forest; OTMFA: Open Tropical Moist Forest with Arecaceae dominance; TMF: Tropical Moist Forest; Cpn: Campinarana.

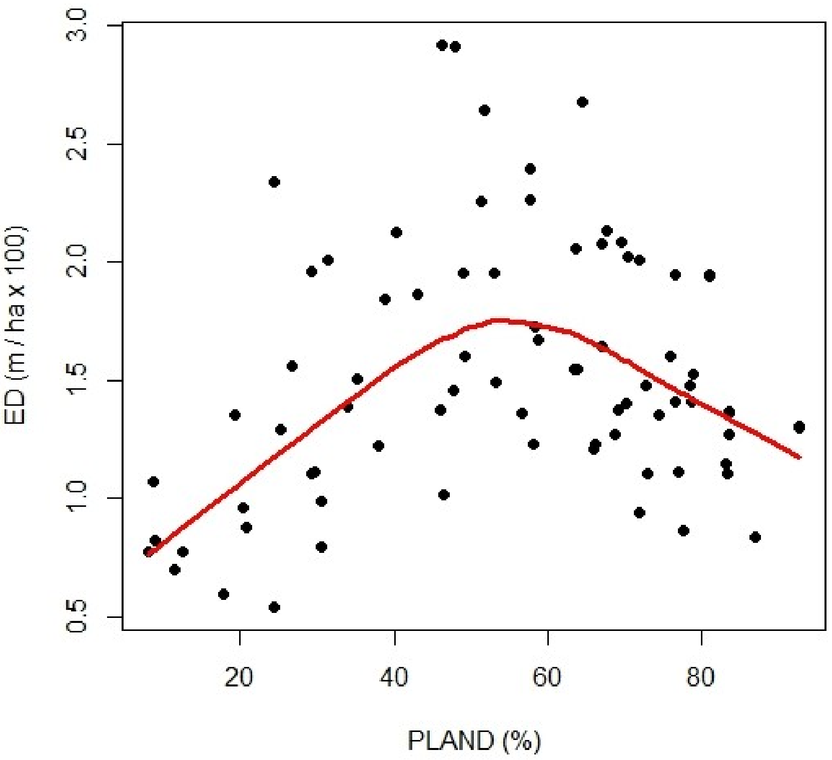

Figure SI 6. Distribution of 79 landscapes samples according to edge density (ED) and percentage of forest cover (PLAND) landscape metrics, that were measured in a subtended circle of 1 km radius with a HLC house in the center. Mosquito collections were in peridomestic habitats across malaria endemic areas of the Brazilian Amazon.

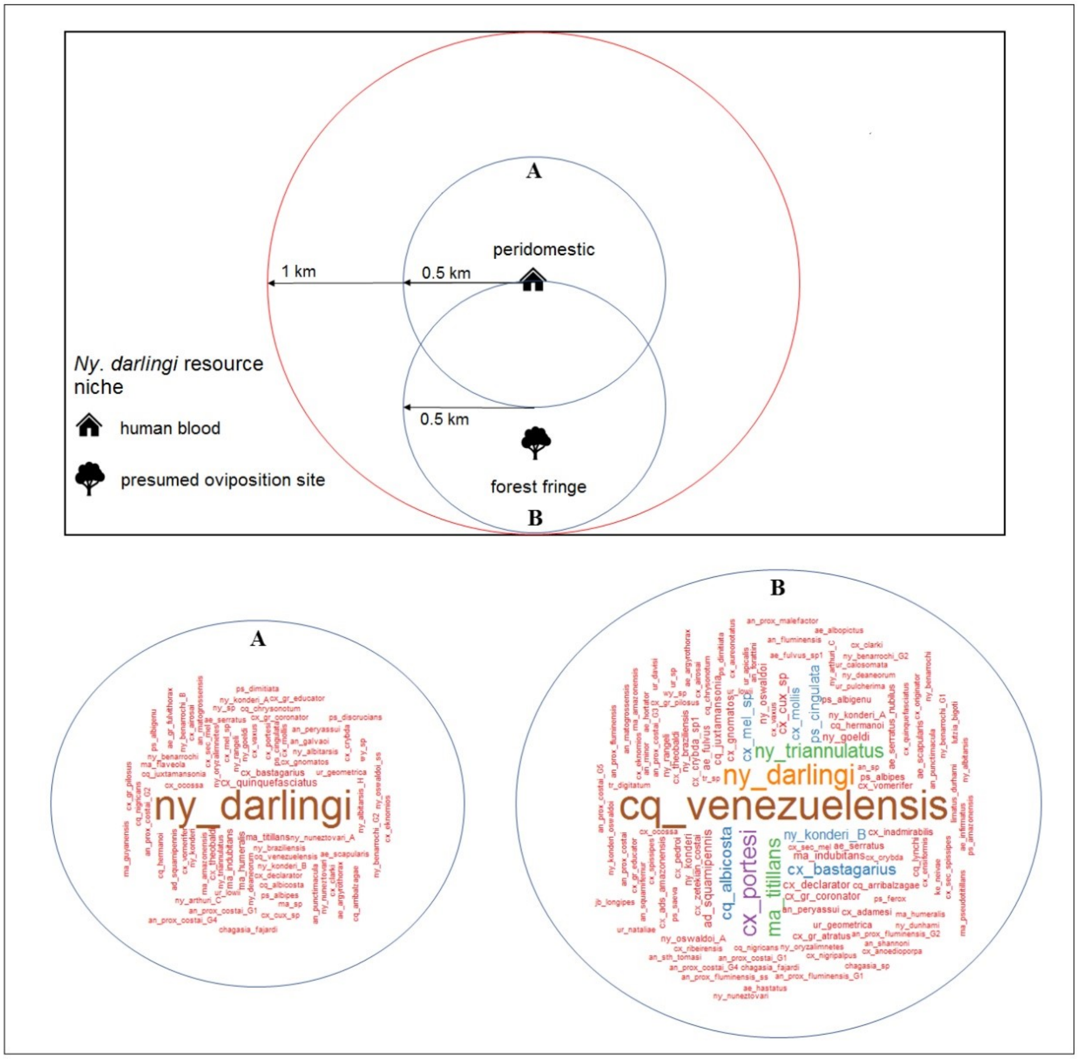

Figure SI 7. Study design adopted to measure the landscape metrics (PLAND, ED, DW), and verify association between landscape structure and *Ny. darlingi* dominance in malaria endemic areas in the Brazilian Amazon, 2015-2017.

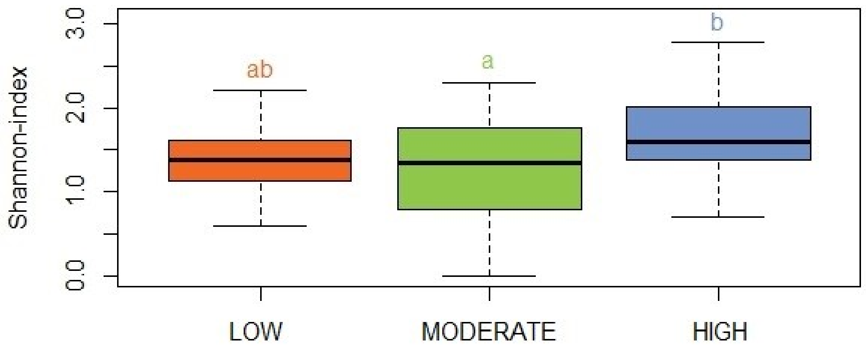

Figure SI 8. Boxplot graph of the Shannon-index values of low (0 – 1.5 m / ha x 100), moderate (1.5 – 2.0 m / ha x 100) and high (2.0 – 3.5 m / ha x 100) edge density in forest fringe habitat. Different colors and letters indicate a significant difference between the treatments by Tukey's test ($P < 0.05$).

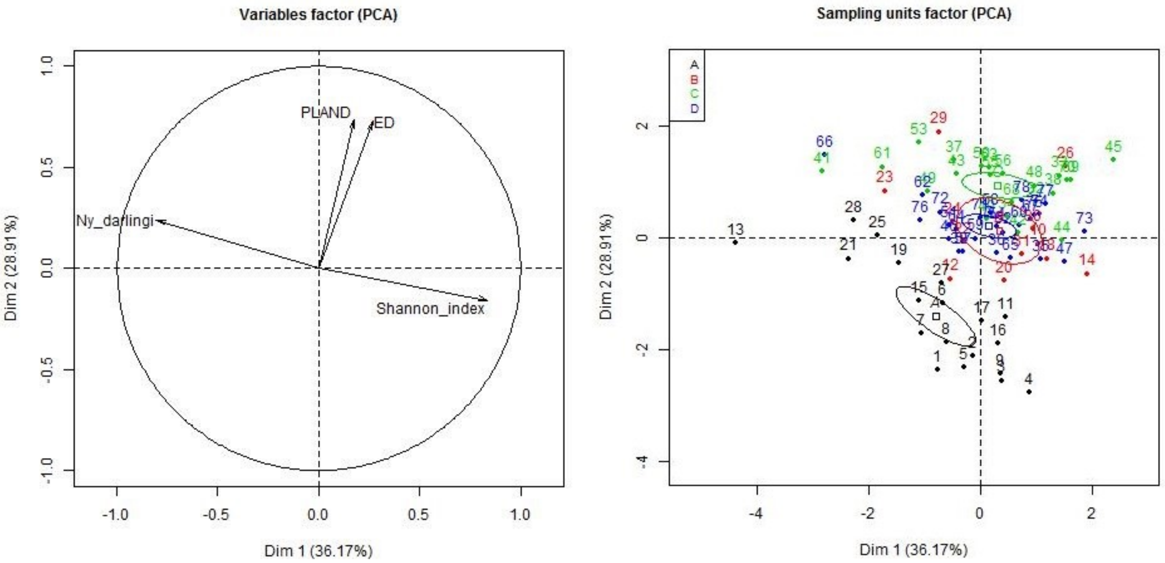

Figure SI 9. PCA graphs of the variables and sampling units' factors showing the variation among *Ny. darlingi* abundance, PLAND, ED and Shannon-index in landscape structure categories: A) open land (PLAND ≤ 50 % and ED < 0.015 m/ha); B) fragmented open land (PLAND ≤ 50 % and ED ≥ 0.015 m/ha); C) fragmented forested land (PLAND > 50 % and ED ≥ 0.015 m/ha); and D) forested land (PLAND > 50 % and ED < 0.015 m/ha).

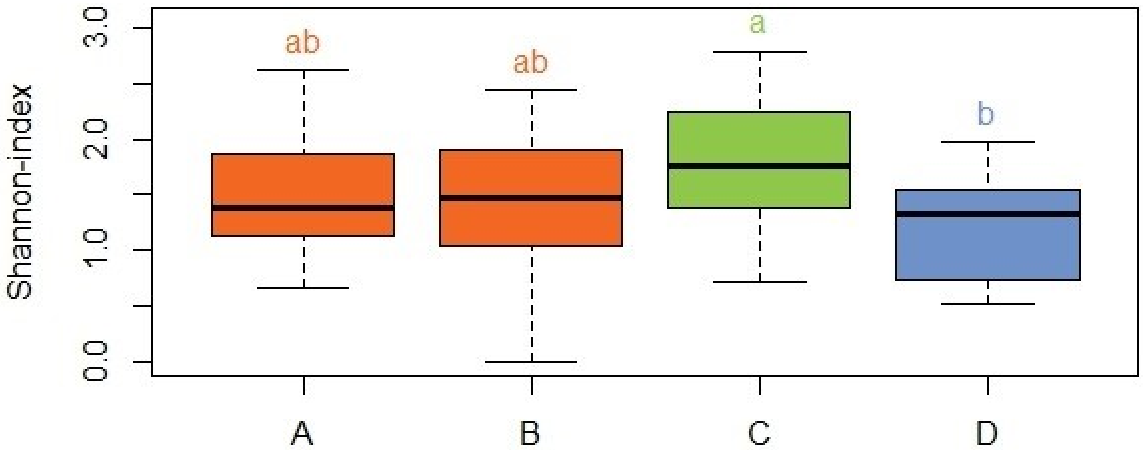

Figure SI 10. Boxplot graph of the Shannon-index values for: A) open land (PLAND ≤ 50 % and ED < 0.015 m/ha); B) fragmented open land (PLAND ≤ 50 % and ED ≥ 0.015 m/ha); C) fragmented forested land (PLAND > 50 % and ED ≥ 0.015 m/ha); and D) forested land (PLAND > 50 % and ED < 0.015 m/ha). Different colors and letters indicate a significant difference between the treatments by Tukey's test ($P < 0.05$).

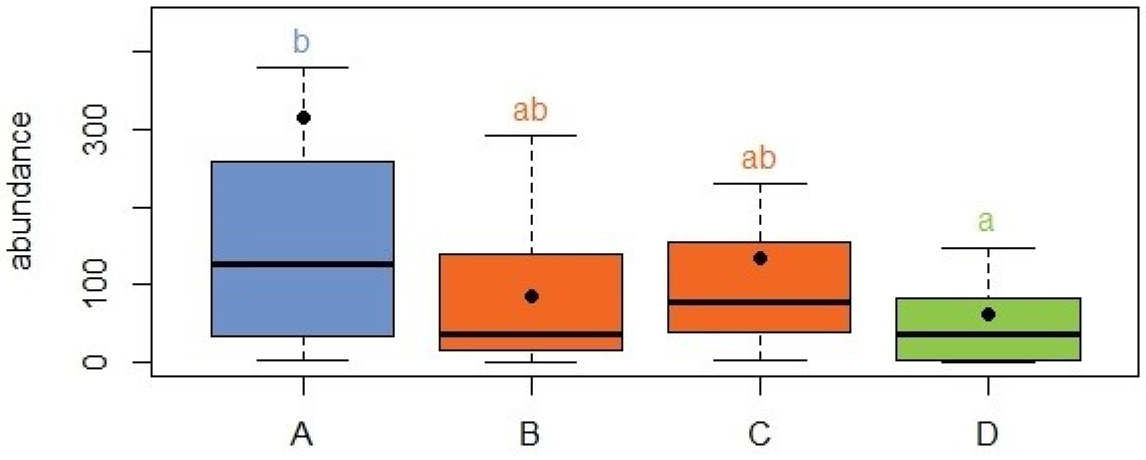

Figure SI 11. Boxplot graph of the *Ny. darlingi* abundance variation in each landscape structure category: A) open land (PLAND ≤ 50 % and ED < 0.015 m/ha); B) fragmented open land (PLAND ≤ 50 % and ED ≥ 0.015 m/ha); C) fragmented forested land (PLAND > 50 % and ED ≥ 0.015 m/ha); and D) forested land (PLAND > 50 % and ED < 0.015 m/ha). Black circles indicate the means. Different colors and letters indicate a significant difference between the treatments by Mann-Whitney-Wilcoxon's multiple comparison test ($P < 0.05$).

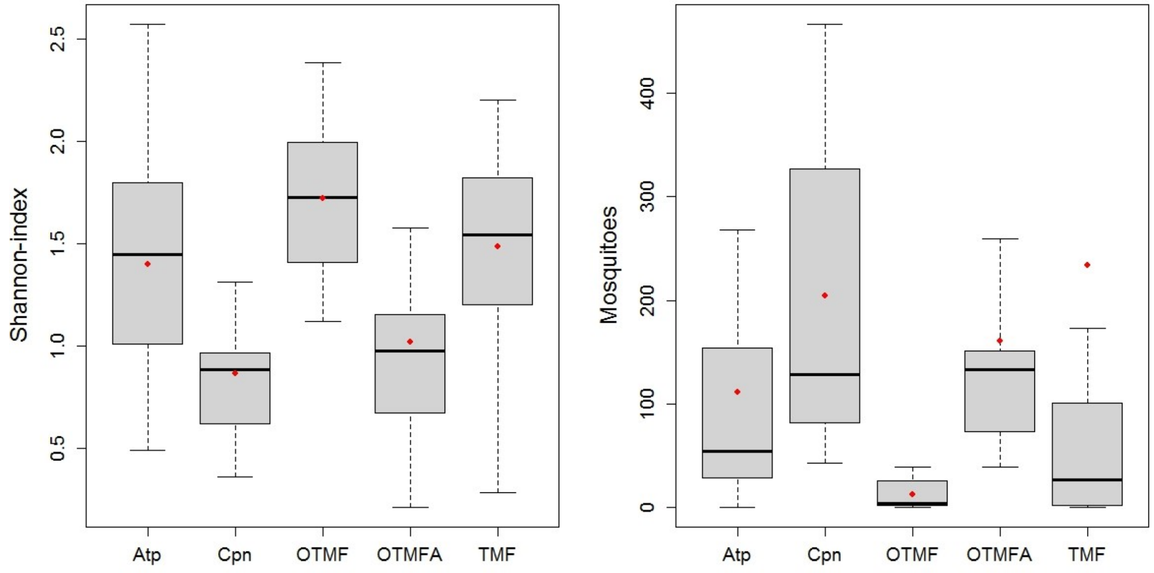

Figure SI 12. Boxplot graph of the Shannon-index variance and *Ny. darlingi* abundance variation according to vegetation class (Brazilian Institute of Geography and Statistics - IBGE classification). Anthropogenic (Atp), Open Tropical Moist Forest (OTMF), Open Tropical Moist Forest with Arecaceae dominance (OTMFA), Tropical Moist Forest (TMF) and Campinarana (Cpn). Red circles indicate the means.

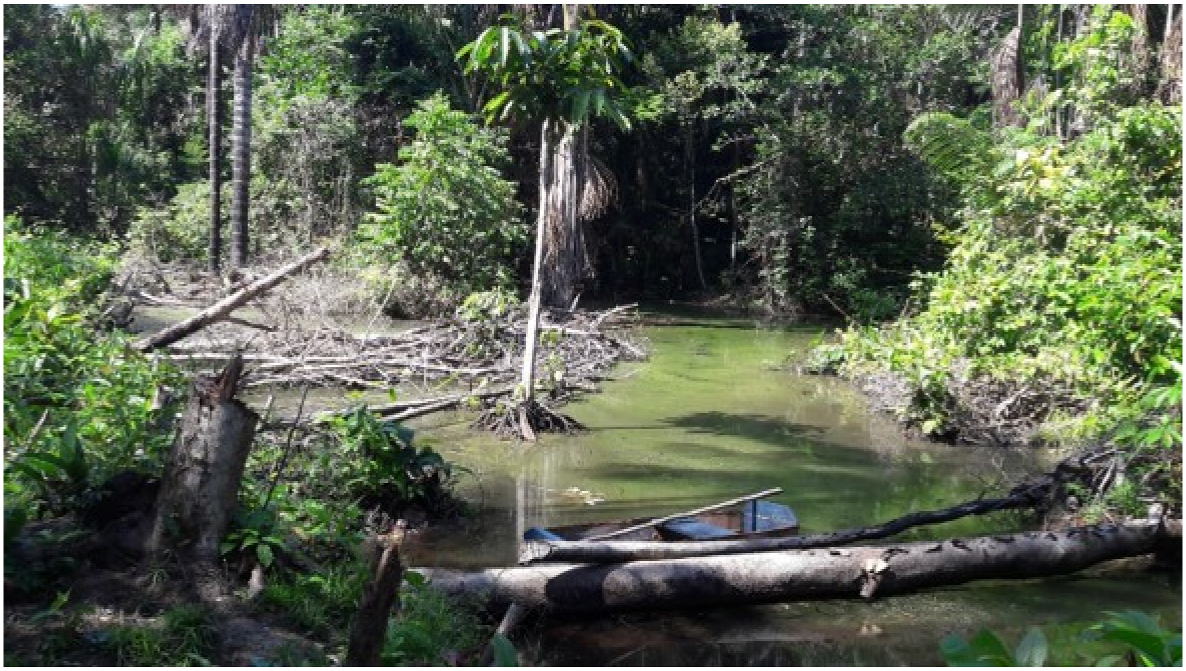

Figure SI 13. Image of the forest fringe habitat with potential pool for *Ny. darlingi* oviposition site / larval habitat. Picture from Presidente Figueiredo, Amazonas field collection. Photo: L.S.M. Chaves, 12th August 2017.

Table SI 4. Path analysis results for *Ny. darlingi* abundance and the independent variables: Shannon-index, PLAND, ED, landscape structure categories (A: PLAND ≤ 50 % and ED < 0.015 m/ha; B: PLAND ≤ 50 % and ED ≥ 0.015 m/ha; C: PLAND > 50 % and ED ≥ 0.015 m/ha; D: PLAND > 50 % and ED < 0.015 m/ha), Amazonian vegetation class (Atp: Anthropogenic; OTMF: Open Tropical Moist Forest; OTMFA: Open Tropical Moist Forest with Arecaceae dominance; TMF: Tropical Moist Forest; Cpn: Campinarana) and DW.

**Regressions**						
Ny. darlingi	**Estimate**	**Standard error**	**Z-value**	**P(>|z|)**	**Standard latent variables**	**Standard all**
Shannon-index	-259.803	74.018	-3.51	0.000	-259.803	-0.407
FC	-266.28	383.109	-0.695	0.487	-266.28	-0.168
ED	690.466	12953.77	0.053	0.957	690.466	0.01
Anthropogenic veg.	-153.879	154.046	-0.999	0.318	-153.879	-0.205
Campinarana	-78.697	169.504	-0.464	0.642	-78.697	-0.069
TMF	43.014	145.932	0.295	0.768	43.014	0.051
OTMFA	-167.648	164.355	-1.02	0.308	-167.648	-0.171
Landscape A	156.604	204.519	0.766	0.444	156.604	0.187
Landscape B	55.371	206.573	0.268	0.789	55.371	0.055
Landscape C	153.804	136.854	1.124	0.261	153.804	0.189
DW	0.002	0.207	0.008	0.994	0.002	0.001
**Shannon-index**						
FC	-0.382	0.581	-0.658	0.511	-0.382	-0.154

ED	-2.12	19.689	-0.108	0.914	-2.12	-0.02
Anthropogenic veg.	-0.434	0.229	-1.897	0.058	-0.434	-0.37
Campinarana	-0.889	0.237	-3.746	0	-0.889	-0.495
TMF	-0.244	0.22	-1.11	0.267	-0.244	-0.183
OTMFA	-0.812	0.233	-3.494	0	-0.812	-0.528
Landscape A	-0.207	0.31	-0.667	0.505	-0.207	-0.158
Landscape B	0.107	0.314	0.342	0.732	0.107	0.068
Landscape C	0.236	0.206	1.145	0.252	0.236	0.186
DW	0.001	0	1.946	0.052	0.001	0.192
**Variances**	**Estimate**	**Std.Err**	**z-value**	**P(>|z|)**	**Std.lv**	**Std.all**
Ny. darlingi	101209.3	16103.57	6.285	0	101209.3	0.764
Shannon-index	0.234	0.037	6.285	0	0.234	0.719
**R-Square**	**Estimate**					
Ny. darlingi	0.236					
Shannon-index	0.281					

SI 5: Link between Amazonian rural settlements and ecological succession.

In Amazonian rural settlements, several ecological succession events may occur simultaneously because the process of land occupation occurs at local and regional scales, with a number of new families beginning the colonization process at various spatiotemporal scales. Stochastic factors such as microenvironmental conditions at the time of human disturbance, the presence of colonialist and opportunist species and larval habitats create conditions for local microecological succession (3). The introduction of both susceptible human populations and *Plasmodium* gametocyte reservoirs as blood sources for *Ny. darlingi* during the lead-up to species dominance, can result in the emergence, reemergence, or intensification of the malaria cycle. The dynamics of human population and behavior in this harsh and unstable environment inevitably facilitate *Plasmodium* propagation, increasing the risk of malaria (4).

The ecological dynamics at forest fringes, areas of forest regeneration and areas of secondary growth provide conditions similar to deforested areas, favoring colonizing species and their dominance in the mosquito community. Previous studies have detected heterogeneity in Culicidae communities in forest edges, forest regeneration, and areas of secondary growth in the Amazon tropical rainforest (5) (6). Interestingly, Connell (7) postulates that a high diversity of trees in tropical rain forest is a non-equilibrium state, therefore, the equilibrium status may never be reached because tropical forests are continuously impacted by disturbances. Gradual changes in climate, landscape structure and continuously disrupted ecological equilibrium, favor an increase in mosquito diversity at the moderate level of forest cover (PLAND = 45 % - 65 %), compared with high and low forest cover. The cleared area represents a suitable habitat for opportunistic species, and

forested landscapes for specialist species. Therefore, the Culicidae richness was similar for both PLAND < 25 % and > 75 %.

High edge density values indicate habitat fragmentation, whereas low values indicate extremes in forest cover percentage. Overlaying the Culicidae diversity onto malaria landscapes metrics, we can measure the strength of the forest disturbance and human land use at different levels, in *Ny. darlingi* life-history strategies, as proposed by Roche and Guégan (8).

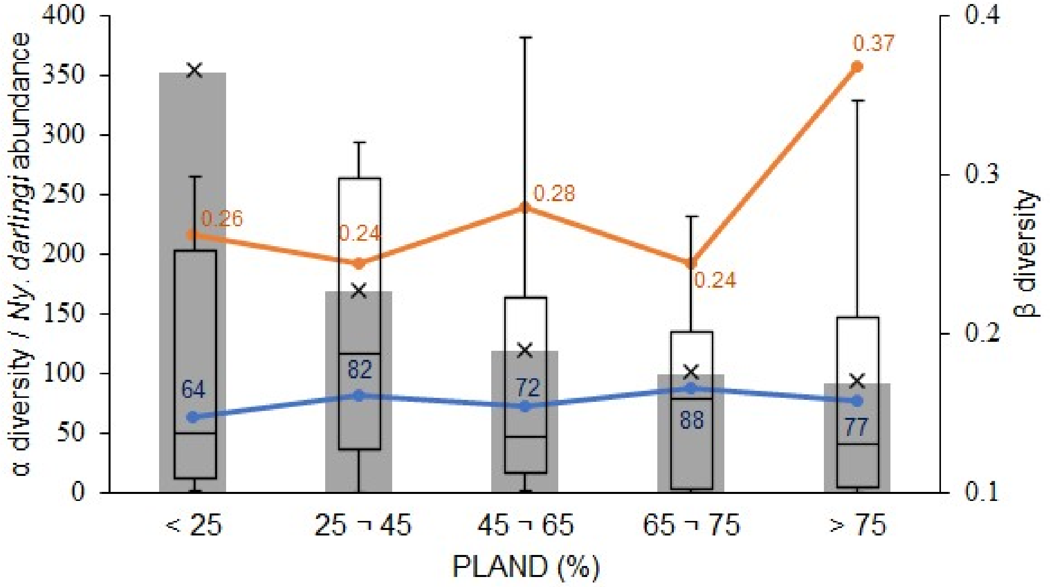

Figure SI 14. Changes in α and β diversity and *Ny. darlingi* distribution according to the forest cover landscape metrics (PLAND) in malaria endemic areas in the Brazilian Amazon. The blue line indicates the α diversity (richness) of Culicidae, the orange line indicates β diversity (turnover) between each category (< 25 %, 25 ¬ 45 %; 45 ¬ 65 %; 65 ¬ 75 % and > 75 %), and grey bars indicate the average numbers of *Ny. darlingi* collected in each PLAND category.

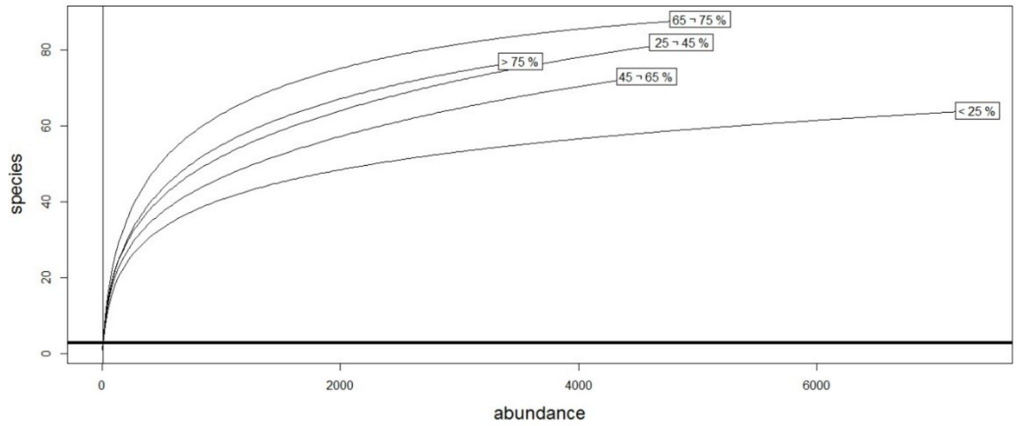

Figure SI 15. Rarefaction curves for five PLAND (%) landscape categories: < 25 %, 25 ¬ 45 %; 45 ¬ 65 %; 65 ¬ 75 % and > 75 %.

Mosquito metabolic activity rates are driven by external energy, where temperature variables are the key for proliferation (9). Therefore, a model projection of a redistribution of predation pressure represented by a high biodiversity richness, can also show where increased threats to global health security are likely to occur under a climate change approach (10), as in global food security (11). Novel biotic communities can result in a cascading effect on biotic interactions with a serious impact on ecosystems and global malaria distribution (12, 13).

Despite not addressing the local dispersal of *Ny. darlingi*, our findings provide some insights about the potential of ecological plasticity of this species in an environment where ecological changes are rapid, continuous and extensive. In addition, environmental pressure

can create conditions to ultimately result in the expansion of *Ny. darlingi* ecological niches (14, 15). Loss of forest cover increases habitat fragmentation, thus increasing sunlight exposure (a strong modulator of anopheline occurrence) and creating new habitats that are promptly occupied by *Ny. darlingi* (16-18). This species has many attributes of an invasive species: intra-population variability, ecological plasticity, environmental change tolerance and interspecific species competition, association with human ecology and high dispersal capacity (19-21). In western Kenya, reducing shade in a larval habitat of *An. gambiae* increased water temperature, thereby reducing the larval-adult development time, increasing survival of the adult population and, consequently, the vectorial capacity, ultimately increasing malaria transmission (22, 23). In the Amazon Basin, such environmental and landscape factors also affect *Ny. darlingi* biting behaviour and peak biting time. In French Guyana, in a high malaria incidence setting, *Ny. darlingi* peaked between 20:30 and 22:30 h at the beginning of the dry season Vezenegho et al. (24), whereas in the localities studied we found that the peaking time ranged depends on forest cover percentage. In landscapes with less than 25 % and between 25 % ¬ 45 % the peaking time was 18-19 h, and in 45 % ¬ 65 %, the species peaked from 20-21h.

Anophelinae diversity can have an important impact on effective vector control strategies in the Amazon River basin. The Amazon forest is species rich for mosquitoes, and includes a large number of undescribed Anophelinae in endemic malaria areas (25) presenting a challenge for surveillance work. Sometimes these species can replace primary vectors, as observed by Conn et al. in Amapá state, Brazil, where *Ny. marajoara* replaced *Ny. darlingi* (Conn et al., 26), or maintain residual transmission that renders intervention actions unsustainable. The replacement of vector species can be a direct or an indirect response to

landscape change. Some studies have investigated a mechanism known as "eco-evolutionary rescue" acting on host-pathogen coexistence. DiRenzo et al.,(27) indicated that an amphibian community in Panama demonstrated stabilizing behavior despite species-specific disease, in a case operating at an ecological time scale. Therefore, the eco-evolutionary process may be happening to *Ny. darlingi* at the landscape scale in the Amazon malaria ecology system. Land use change may have the effect of stabilizing mosquito communities in these areas by favoring species with high ecological plasticity.

Therefore, high local biodiversity can be a protective factor when equilibrium is reached between the host-vector interactions and a multi-trophic chain, representing health protection. In this case, biodiversity loss can be a predictor of increasing malaria incidence in malaria endemic areas (28). Furthermore, in a recent meta-analysis study Civitello et al. (29) show that biodiversity can inhibit the spread of parasites because of the dilution effect mechanisms.

Literature Cited

1. IBGE – Brazilian Institute of Geography and Statistics. Manual técnico da vegetação brasileira. Manuais técnicos em geociências. 2012;1.

2. McGarigal K, Marks BJ. FRAGSTATS: spatial pattern analysis program for quantifying landscape structure. Gen Tech Rep PNW-GTR-351 Portland, OR: US Department of Agriculture, Forest Service, Pacific Northwest Research Station 122 p. 1995;351.

3. Loaiza JR, Dutari LC, Rovira JR, Sanjur OI, Laporta GZ, Pecor J, et al. Disturbance and mosquito diversity in the lowland tropical rainforest of central Panama. Scientific reports. 2017;7(1):7248.

4. Ferreira MU, Castro MC. Challenges for malaria elimination in Brazil. Malaria journal. 2016;15(1):284.

5. Djènontin A, Bio-Bangana S, Moiroux N, Henry M-C, Bousari O, Chabi J, et al. Culicidae diversity, malaria transmission and insecticide resistance alleles in malaria vectors in Ouidah-Kpomasse-Tori district from Benin (West Africa): A pre-intervention study. Parasites & vectors. 2010;3(1):83.

6. Hutchings RSG, Sallum MAM, Hutchings RW. Mosquito (Diptera: Culicidae) diversity of a forest-fragment mosaic in the Amazon rain forest. Journal of medical entomology. 2011;48(2):173-87.

7. Connell JH. Diversity in tropical rain forests and coral reefs. Science. 1978; 199 (4335): 1302-10.

8. Roche B, Guegan J-F. Ecosystem dynamics, biological diversity and emerging infectious diseases. Comptes rendus biologies. 2011; 334(5-6): 385-92.

9. Neven LG. Physiological responses of insects to heat. Postharvest Biology and Technology. 2000; 21(1): 103-11.

10. Romero GQ, Gonçalves-Souza T, Kratina P, Marino NA, Petry WK, Sobral-Souza T, et al. Global predation pressure redistribution under future climate change. Nature Climate Change. 2018;8(12):1087.

11. Wheeler T, Von Braun J. Climate change impacts on global food security. Science. 2013; 341(6145): 508-13.

12. Pecl GT, Araújo MB, Bell JD, Blanchard J, Bonebrake TC, Chen I-C, et al. Biodiversity redistribution under climate change: Impacts on ecosystems and human well-being. Science. 2017;355(6332):eaai9214.

13. Caminade C, Kovats S, Rocklov J, Tompkins AM, Morse AP, Colón-González FJ, et al. Impact of climate change on global malaria distribution. Proceedings of the National Academy of Sciences. 2014;111(9):3286-91.

14. Garrido-Garduño T, Téllez-Valdés O, Manel S, Vázquez-Domínguez E. Role of habitat heterogeneity and landscape connectivity in shaping gene flow and spatial population structure of a dominant rodent species in a tropical dry forest. Journal of Zoology. 2016;298(4):293-302.

15. Cote J, Bestion E, Jacob S, Travis J, Legrand D, Baguette M. Evolution of dispersal strategies and dispersal syndromes in fragmented landscapes. Ecography. 2017; 40(1): 56-73.

16. de Barros FSM, Honorio NA, Arruda ME. Survivorship of *Anopheles darlingi* (Diptera: Culicidae) in relation with malaria incidence in the Brazilian Amazon. PloS one. 2011;6(8):e22388.

17. Sanchez-Mazas A, Cerny V, Di D, Buhler S, Podgorna E, Chevallier E, et al. The HLA-B landscape of Africa: Signatures of pathogen-driven selection and molecular identification of candidate alleles to malaria protection. Mol Ecol. 2017.

18. Sánchez-Ribas J, Oliveira-Ferreira J, Gimnig JE, Pereira-Ribeiro C, Santos-Neves MSA, Silva-do-Nascimento TF. Environmental variables associated with anopheline larvae distribution and abundance in Yanomami villages within unaltered areas of the Brazilian Amazon. Parasites & vectors. 2017;10(1):571.

19. Hiwat H, Bretas G. Ecology of *Anopheles darlingi* Root with respect to vector importance: a review. Parasites & vectors. 2011;4(1):177.

20. Manni M, Guglielmino CR, Scolari F, Vega-Rúa A, Failloux A-B, Somboon P, et al. Genetic evidence for a worldwide chaotic dispersion pattern of the arbovirus vector, *Aedes albopictus*. PLoS neglected tropical diseases. 2017; 11(1): e0005332.

21. Juliano SA, Philip Lounibos L. Ecology of invasive mosquitoes: effects on resident species and on human health. Ecology letters. 2005;8(5):558-74.

22. Afrane YA, Lawson BW, Githeko AK, Yan G. Effects of microclimatic changes caused by land use and land cover on duration of gonotrophic cycles of *Anopheles gambiae* (Diptera: Culicidae) in western Kenya highlands. Journal of medical entomology. 2005; 42(6): 974-80.

23. Afrane YA, Zhou G, Lawson BW, Githeko AK, Yan G. Effects of microclimatic changes caused by deforestation on the survivorship and reproductive fitness of *Anopheles gambiae* in western Kenya highlands. The American journal of tropical medicine and hygiene. 2006;74(5):772-8.

24. Vezenegho SB, Adde A, Santi VPd, Issaly J, Carinci R, Gaborit P, et al. High malaria transmission in a forested malaria focus in French Guiana: How can exophagic *Anopheles darlingi* thwart vector control and prevention measures? Memórias do Instituto Oswaldo Cruz. 2016;111(9):561-9.

25. Bourke BP, Conn JE, de Oliveira TMP, Chaves LSM, Bergo ES, Laporta GZ, et al. Exploring malaria vector diversity on the Amazon Frontier. Malar J. 2018; 17(1): 342.

26. Conn JE, Wilkerson RC, Segura MN, de Souza RT, Schlichting CD, Wirtz RA, et al. Emergence of a new neotropical malaria vector facilitated by human migration and changes in land use. Am J Trop Med Hyg. 2002; 66(1): 18-22.

27. DiRenzo GV, Zipkin EF, Grant EHC, Royle JA, Longo AV, Zamudio KR, et al. Eco-evolutionary rescue promotes host–pathogen coexistence. Ecological Applications. 2018; 28(8): 1948-62.

28. Keesing F, Belden LK, Daszak P, Dobson A, Harvell CD, Holt RD, et al. Impacts of biodiversity on the emergence and transmission of infectious diseases. Nature. 2010; 468(7324): 647.

29. Civitello DJ, Cohen J, Fatima H, Halstead NT, Liriano J, McMahon TA, et al. Biodiversity inhibits parasites: broad evidence for the dilution effect. Proceedings of the National Academy of Sciences. 2015; 112(28): 8667-71.
